# The Effects of Exergaming on Executive and Physical Functions in Older Adults With Dementia: Randomized Controlled Trial

**DOI:** 10.2196/39993

**Published:** 2023-03-07

**Authors:** Shanshan Wu, Hongqing Ji, Junyeon Won, Eun-Ah Jo, Yun-Sik Kim, Jung-Jun Park

**Affiliations:** 1 School of Physical Education & Health Wenzhou University Wenzhou China; 2 Division of Sport Science Pusan National University Busan Republic of Korea; 3 Institute for Exercise and Environmental Medicine Texas Health Presbyterian Hospital Dallas, TX United States; 4 Department of Internal Medicine Kosin University College of Medicine Busan Republic of Korea

**Keywords:** exergame, exergaming, executive function, physical function, reaction time, N2, P3b, physical, function, game, dementia, RCT, cognitive function, older adults, aerobic exercise, exercise, neuronal, activity, task, stimulation, intervention

## Abstract

**Background:**

Despite increasing interest in the effects of exergaming on cognitive function, little is known about its effects on older adults with dementia.

**Objective:**

The purpose of this is to investigate the effects of exergaming on executive and physical functions in older adults with dementia compared to regular aerobic exercise.

**Methods:**

In total, 24 older adults with moderate dementia participated in the study. Participants were randomized into either the exergame group (EXG, n=13, 54%) or the aerobic exercise group (AEG, n=11, 46%). For 12 weeks, EXG engaged in a running-based exergame and AEG performed a cycling exercise. At baseline and postintervention, participants underwent the Ericksen flanker test (accuracy % and response time [RT]) while recording event-related potentials (ERPs) that included the N2 and P3b potentials. Participants also underwent the senior fitness test (SFT) and the body composition test pre- and postintervention. Repeated-measures ANOVA was performed to assess the effects of time (pre- vs postintervention), group (EXG vs AEG), and group×time interactions.

**Results:**

Compared to AEG, EXG demonstrated greater improvements in the SFT (*F*_1.22_=7.434, *P*=.01), reduction in body fat (*F*_1.22_=6.476, *P*=.02), and increase in skeletal mass (*F*_1.22_=4.525, *P*=.05), fat-free mass (*F*_1.22_=6.103, *P*=.02), and muscle mass (*F*_1.22_=6.636, *P*=.02). Although there was a significantly shorter RT in EXG postintervention (congruent *P*=.03, 95% CI 13.581-260.419, incongruent *P*=.04, 95% CI 14.621-408.917), no changes occurred in AEG. EXG also yielded a shorter N2 latency for central (Cz) cortices during both congruent conditions compared to AEG (*F*_1.22_=4.281, *P*=.05). Lastly, EXG presented a significantly increased P3b amplitude compared to AEG during the Ericksen flanker test (congruent: frontal [Fz] *F*_1.22_=6.546, *P*=.02; Cz *F*_1.22_=5.963, *P*=.23; parietal [Pz] *F*_1.22_=4.302, *P*=.05; incongruent: Fz *F*_1.22_=8.302, *P*=.01; Cz *F*_1.22_=15.199, *P*=.001; Pz *F*_1.22_=13.774, *P*=.001).

**Conclusions:**

Our results suggest that exergaming may be associated with greater improvements in brain neuronal activity and enhanced executive function task performance than regular aerobic exercise. Exergaming characterized by both aerobic exercise and cognitive stimulation can be used as an effective intervention to improve cognitive and physical functions in older adults with dementia.

**Trial Registration:**

Clinical Research Information Service KCT0008238; https://cris.nih.go.kr/cris/search/detailSearch.do/24170

## Introduction

Executive function is a family of at least 3 different functions that include the maintenance and execution of target tasks by suppressing interference factors during information processing [1]. Execution function is not a single structure but can be divided into (1) a core (inhibition, renewal/working memory, and conversion) and (2) a higher level (planning/solving problems) that depends heavily on the frontal lobes [2,3]. Executive dysfunction often represents age-related cognitive decline and dementia, which are also associated with a declining ability to independently maintain activities of daily living [4-6]. Indeed, dementia-related deficits in executive function cause fatal impairment in activities in daily living in older adults [7,8].

Neural activity during executive function tasks can be measured using electroencephalography (EEG) to record event-related potentials (ERPs) during neuropsychological task performance. The Eriksen flanker test, the Stroop task, the Go/No-go task, and Trail Making Test B are commonly used in neuropsychological test batteries to assess executive function. In the ERP component, N2 is a negative potential that appears 200 ms after stimulation, while P3b is a positive potential that appears 300-600 ms after stimulation [9,10]. N2 is a negative peak before P3b and is related to the measurement of executive function and attention [11]. P3b reflects the update of working memory [12]. It has been known that older adults with dementia have a smaller N2 amplitude [11], a longer N2 latent period [13], and a smaller P3b amplitude, as well as a longer P3b latent phase [14,15], compared to their cognitively intact counterparts.

Previous studies have reported that cognitive training and exercise have salutary effects on the improvement of executive function [16-19]. For example, cognitive training promotes enhanced executive function, learning ability, and memory [20]. In addition, both acute and chronic exercises have beneficial effects on executive function and neural efficiency in older adults [19,21-23]. Despite evidence linking cognitive and exercise training with neurocognitive health, few studies have evaluated the joint effects of cognitive and exercise training in older individuals with dementia. Exergaming has the characteristics of both cognitive (ie, auditory and visual stimulation) and exercise training (ie, motor-sensory stimulation) [24,25]. However, the effects of exercise on cognitive and brain function have been mainly tested in older adults with intact cognition. Thus, little is known about the effects manifested by exergaming on older adults with dementia.

In addition, previous studies using exergaming have shown that exergaming not only improves executive function and cognitive processing but also reduces the risk of falls in older adults [26-30]. Nevertheless, previous findings focused on older adults with intact cognition; thus, the effects of exergaming on older adults with dementia remain largely unexplored. Moreover, little is known about the effects of exergaming on the neural responses during executive function in older adults with dementia. Evidence suggests that virtual reality–based exercise intervention increases P3 and N2 amplitudes and decreases N2 latency, suggesting improvement in cognitive processing in older adults [31]. Furthermore, exercise provides beneficial effects in reducing the risk of decreased physical function, such as balance [32-34], gait [34], and mobility [35], and poor muscular power and functional capacity [32,36]. Given the exercise training component of exergaming (ie, aerobic and strength capacities), we postulated that exergaming may be effective in not only cognitive function but also in physical function. Hence, this study investigated the effects of exergaming on cognitive and physical functions in older adults with dementia. We also compared the effects of exergaming and traditional forms of aerobic exercise on cognitive and physical functions.

## Methods

### Participants

We recruited 52 sedentary older male and female adults with mild or moderate dementia from 2 daycare centers in Busan, South Korea. All participants were diagnosed with dementia determined by their primary care physician and had comorbidities, such as diabetes and hypertension, prior to participating in the study. Inclusion criteria were as follows: (1) age≥65 years, (2) Korean Mini Mental State Examination (MMSE-K) score=15-23, (3) diagnosis of probable dementia based on comprehensive neuropsychological testing (Korean Consortium to Establish a Registry for Alzheimer’s Disease [CERAD-K] test battery), and (4) ability to perform physical exercise and daily life (Korean Activity of Daily Living [K-ADL]).

### Sample Size

**Figure 1:**
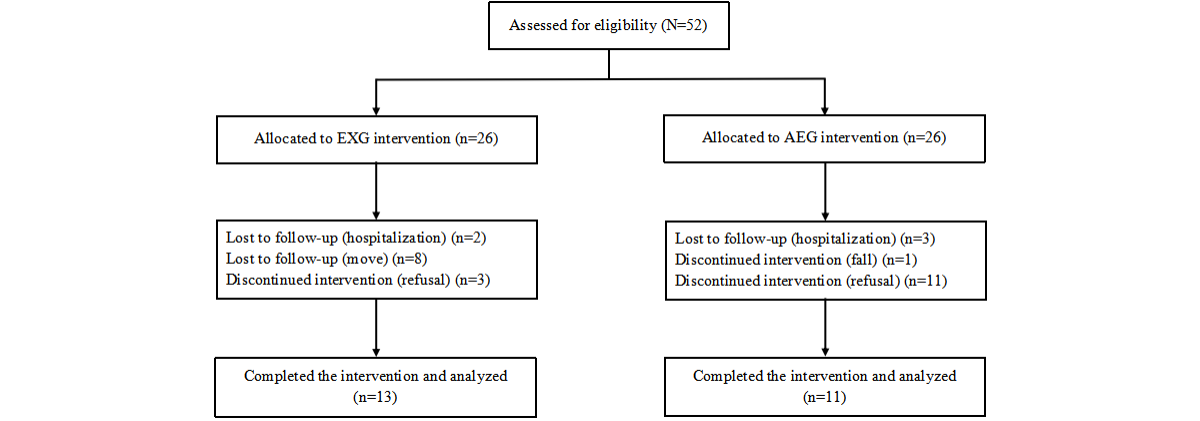
Flowchart of participant eligibility, withdrawals, and the final sample included in the final analysis.

Initially, the study population comprised 52 participants, who were randomly assigned to 2 groups: the exergame group (EXG, n=26, 50%) and the aerobic exercise group (AEG, n=26, 50%); see Figure 1. However, 13 (50%) EXG participants were lost to follow-up at 4 weeks (2, 15%, hospitalizations; 8, 62%, moves to other daycare centers; and 3, 23%, refusals to exercise). From AEG, 15 (58%) participants dropped out during the intervention (3, 20%, hospitalizations; 1, 7%, fall; and 11, 73%, refusals to exercise). Therefore, the data of 13 (50%) EXG participants and 11 (42%) AEG participants, who completed the 12-week exercise program, were included in the data analysis for this study.

The sample size was calculated using a sample size calculation software program (G*Power version 3.1.9.2 for Windows), with an effect size of 0.58, a statistical power of 0.80, and a statistical level of significance of .05. The effect size was calculated from previous studies [37]. As a result, the sample size for each group was established at 20 patients, so we decided to recruit 52 patients for each group, considering a potential 30% dropout rate.

### Ethical Considerations

All participants completed a written informed consent form approved by the Institutional Review Board of Pusan National University (PNU IRB/2018_59_HR).

### Design

This study was a single-blind randomized controlled trial, which was conducted at 2 daycare centers in Busan, South Korea. Randomization was administered by a person who was not involved in this study using computer-generated random numbers. Outcome measurements were performed pre- and postintervention by a blinded assessor with 3 months of clinical experience.

### Exercise Training Interventions

**Figure 2:**
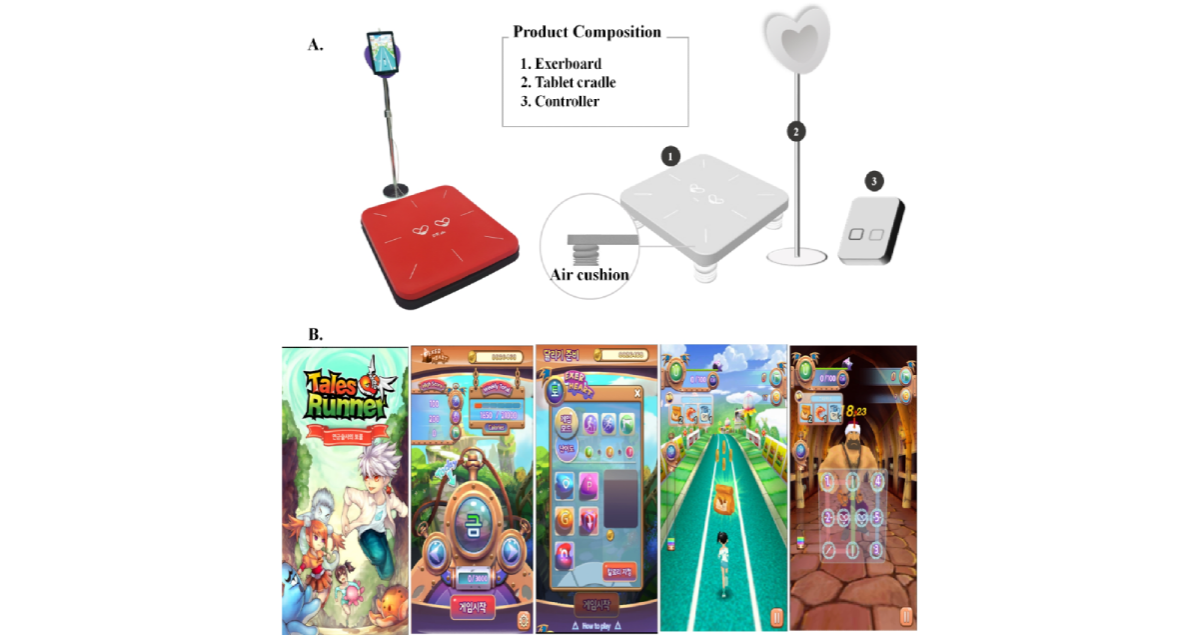
(A) EXG performed the exercise using ExerHeart devices by permission of D&J Humancare, which is the copyright holder of ExerHeart. (B) Features of the video game Alchemist's Treasure. EXG: exergame group.

All participants in both EXG and AGE underwent 2 weeks of a familiarization period before starting the 12-week intervention. The frequency of both exergaming and aerobic exercise was 3 days/week. The intensity of both groups was 60%-70% of the heart rate reserved (HRR) and was gradually increased (ie, 30 minutes for weeks 1-2, 35 minutes for week 3, 40 minutes for weeks 4-5, 45 minutes for weeks 6-7, and 50 minutes for weeks 8-12). Table 1 presents the details of the exercise protocol. All participants’ heart rates (HRs) were measured during exercise using HR monitors (Polar RS400sd, Madison Height, MI, USA). The HRR was calculated using the Karvonen formula [38]. EXG performed aerobic exercise using ExerHeart devices (D&J Humancare, Busan, South Korea) equipped with a running/jumping mat (950 width × 1300 depth × 1700 height); see Figure 2A. ExerHeart is an intelligent exercise management service developed by experts in medical and exercise science. We used a game called *Alchemist's Treasure*, which is a running game based on the Talesrunner IP co-developed with ExerHeart (Figure 2B). During the game, players run with the avatar, avoiding obstacles, and win items while running or jumping at speed on the mat (Multimedia Appendix 1).

AEG performed a cycling exercise using the commercial recumbent cycle EGOJIN 704 (China). The initial bicycle resistance was 0 Kp, and the exercise intensity was increased by 1 Kp every 5 minutes. Different resistance values were used for males and females during exercise to account for the sex-related difference in cardiorespiratory fitness and lower body strength (eg, males: weeks 1-3 up to 3 Kp, weeks 4-12 up to 4 Kp; females: weeks 1-3 up to 2 Kp, weeks 4-12 up to 3 Kp).

**Table 1:** Exergame and aerobic exercise program.

Order and type		Time (minutes)	Workload (Kp)
**Warm-up**			
	Stretching (neck, shoulder, arm, wrist, waist, leg, ankle)	10	N/A^a^
**Main exercise (EXG^b^); HRR^c^=60%-65%, frequency=3 days/week **			
	Phase Ⅰ (week 1): exercise × 2 times (5 minutes each)	10	N/A
	Phase Ⅱ (week 2): exercise	10	N/A
	Phase Ⅲ (week 3): exercise	15	N/A
	Phase Ⅳ (weeks 4-5): exercise	20	N/A
	Phase Ⅴ (weeks 6-7): exercise	25	N/A
	Phase Ⅵ (weeks 8-12): exercise	30	N/A
**Main** **exercise (AEG^d^); HRR^c^=60%-65%, frequency=3 days/week**			
	Phase Ⅰ (week 1): exercise × 2 times (5 minutes each)	10	0-3
	Phase Ⅱ (week 2): exercise	10	0-3
	Phase Ⅲ (week 3): exercise	15	0-3
	Phase Ⅳ (weeks 4-5): exercise	20	2-4
	Phase Ⅴ (weeks 6-7): exercise	25	2-4
	Phase Ⅵ (weeks 8-12): exercise	30	2-4
**Cool down**			
	Stretching (neck, shoulder, arm, wrist, waist, leg, ankle)	N/A	N/A

^a^N/A: not applicable.

^b^EXG: exergame group.

^c^HRR: heart rate reserved.

^d^AEG: aerobic exercise group.

### Outcome Measures

#### Eriksen Flanker Test

A modified version of the Eriksen flanker test was used to assess executive function [39]. We used the computerized Eriksen flanker test using the built-in software of the EEG analyzer (Telescan). Participants were positioned about 1 m in front of the computer screen. Each trial began with a 1500-9500 ms fixation blank screen, followed by a precue (500 ms) consisting of dashed lines to indicate that the stimulus was about to appear. Each flanker stimulus was presented for 500 ms, followed by a 1500 ms response window. In each trial, participants were instructed to press buttons with the left or right index finger in the direction of a centrally positioned target arrow as quickly and accurately as possible. The central arrow (called the target) was always flanked by a set of 2 irrelevant arrows on each side (called the flankers); the target and flankers pointed either in the same direction (congruent signal, “< < < < <” or “> > > > >”) or in opposite directions (incongruent signal, “> > < > >” or “< < > < <”). A total of 280 targets consisting of 140 interstimulus intervals, 80 congruent signals, and 60 incongruent signals were presented to each participant. The Eriksen flanker test was conducted before and after the 12-week exercise intervention.

#### Electroencephalographic Measurements

EEG activity was recorded during the modified Eriksen flanker test using a computerized polygraph system with a 31-channel Poly G-A (LAXTHA, Daejeon, South Korea). Ag-AgCl electrodes were placed on frontal (Fz), central (Cz), and parietal (Pz) cortex areas, according to the International 10-20 system. Midline locations were referenced to link earlobe electrodes. Horizontal and vertical electrooculograms were monitored by electrodes placed above and below the left eye and at the outer canthus of both eyes, respectively. The impedance of all electrodes was maintained below 10 kΩ. The bandpass filter of the amplifier was 0.3-30 Hz, the sampling rate was 1000 Hz, and a notch filter was at 60 Hz.

The stimulus-locked epochs acquired for the Eriksen flanker test were extracted offline from 200 ms before and ending 1500 ms after the stimulus onset, and the period from –100 to 0 ms before stimuli onset was used as the baseline. Peak amplitudes and latencies were measured automatically. The N2 and P3b peak amplitudes and latencies were measured in the difference waves of the attended condition. The N2 component was defined as the largest positive peak occurring between 180 and 315 ms after stimuli, and the P3b component was defined as the largest positive peak occurring between 300 and 600 ms after stimuli. EEG data were collected before and after the 12-week exercise intervention.

#### Senior Fitness Test

The senior fitness test (SFT) consists of 5 items: (1) upper and (2) lower body muscle strength test (arm curl test/30-second chair stand test), (3) upper and (4) lower body flexibility test (back scratch test/chair sit-and-reach test), and (5) cardiopulmonary endurance test (2-minute step-in-place test). The SFT items used in this study were as follows:

- Arm curl test: counting the number of full curls in 30 seconds, holding a 3.6 kg weight on the hand for men and 2.3 kg for women
- 30-second chair stand test: counting the number of full stands in 30 seconds with arms folded across the chest
- Back scratch test: assessing whether one hand can reach over the shoulder and the other hand in the middle of the back and measuring the distance in centimeters (+ or –) between extended middle fingers
- Chair sit-and-reach test: performing a sitting pose in front of a chair, with 1 leg extended and hands reaching as far as possible toward the toes, and measuring the distance in centimeters (+ or –) between extended fingers and the tips of the toes
- 2-minute step-in-place test: standing up straight next to a wall, while a mark is placed on the wall at a level corresponding to midway between the patella (knee cap) and iliac crest (top of the hip bone), marching in place for 2 minutes by lifting the knees to the height of the mark on the wall and counting the total number of times the right knee reaches the tape level.

The SFT was administered before and after the 12-week exercise intervention to examine physical fitness.

#### Body Composition

The body composition in terms of the BMI, percentage of body fat, bone mass, fat-free mass, and muscle mass was measured using the bioelectrical impedance analysis (BIA) system InBody S10 (Seoul Biospace Ltd., Korea). These measurements were performed after participants rested in the supine position for 10 minutes. All participants did not eat or exercise for 3 hours before the test and did not drink alcohol or caffeine for 24 hours.

### Statistical Analysis

We performed per protocol analyses using linear random effects models for each outcome measure. We performed the Shapiro-Wilk test to assess whether the data were normally distributed. The Eriksen flanker test (ie, accuracy rate and response time [RT]) and ERP (ie, N2, P3b amplitudes) data were analyzed using repeated-measures ANOVA to determine group (EXG vs AEG) × time (pre- vs postintervention) interactions. We also used the paired *t* test (or Wilcoxon signed-rank test) to examine the changes of each dependent variable after the intervention within each group. Bonferroni post hoc analyses were performed when there was a significant difference. Partial *η*^2^_p_ was used to assess the effect size. The statistical significance was set at α=.05. All statistical tests were conducted using IBM SPSS Statistics (version 24.0).

## Results

### Participants

Of 52 participants, 13 (25%) patients in EXG (n=5, 38%, males and n=8, 62%, females) and 11 (21%) patients in AEG (n=3, 27%, males and n=8, 73%, females) completed the 12-week exercise program. Demographic and physical characteristics of all subjects are provided in Table 2. Although there was a significantly greater body fat percentage in AEG compared to EXG at baseline, no other group differences were found in baseline measurements.

**Table 2:** Baseline characteristics of demographic information.

Variables		EXG^a^ (n=13)	AEG^b^ (n=11)	Group differences (*P* value)
**Demographics**				
	Gender ratio, male:female	5:8	3:8	N/A^c^
	Age (years), mean (SD)	78.8 (4.8)	81.2 (4.4)	.22
	Height (cm), mean (SD)	159.0 (9.1)	154.0 (6.0)	.13
	Weight (kg), mean (SD)	59.8 (6.8)	59.3 (8.0)	.86
	MMSE-K^d^ score, mean (SD)	17.8 (3.7)	18.8 (2.5)	.43
	K-GDS^e^ score, mean (SD)	7.1 (5.1)	7.9 (6.8)	.74
	K-ADL^f^ score, mean (SD)	9.1 (1.7)	8.4 (1.5)	.32
**CERAD-K^g^, mean (SD)**				
	Verbal fluency test score	7.5 (3.8)	6.2 (3.3)	.36
	Modified Boston naming test score	6.3 (3.7)	6.5 (2.4)	.91
	Word list memory test score	8.4 (4.3)	7.5 (4.5)	.60
	Constructional praxis test score	6.6 (3.2)	7.3 (2.1)	.56
	Word list recall test score	1.1 (1.3)	0.5 (1.2)	.25
	Word list recognition test score	3.2 (2.7)	3.6 (3.0)	.69
	Constructional recall test score	1.8 (2.0)	0.6 (1.1)	.11
**Body composition** **, mean (SD)**				
	BMI (kg/m^2^)	23.0 (1.6)	25.0 (3.0)	.06
	Body fat percentage (%)	36.7 (5.0)	43.1 (5.6)	.01
	Skeletal mass (kg)	19.7 (3.2)	17.3 (2.6)	.07
	Fat-free mass (kg)	37.7 (5.5)	33.5 (4.4)	.08
	Muscle mass (kg)	35.5 (5.3)	31.6 (4.2)	.09

^a^EXG: exergame group.

^b^AEG: aerobic exercise group.

^c^N/A: not applicable.

^d^MMSE-K: Korean Mini Mental State Examination.

^e^K-GDS: Korean Geriatric Depression Scale.

^f^K-ADL: Korean Activity of Daily Living.

^g^CERAD-K: Korean Consortium to Establish a Registry for Alzheimer’s Disease.

### Eriksen Flanker Test Performance

**Figure 3:**
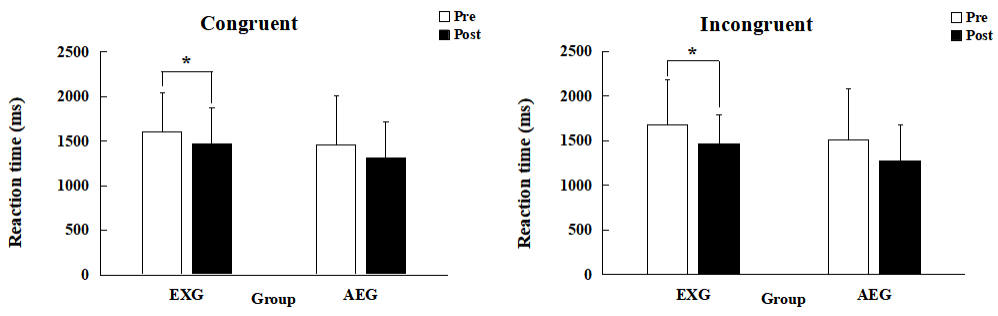
RT during the Eriksen flanker test performance before and after the 12-week exercise intervention: (A) congruent and (B) incongruent. AEG: aerobic exercise group; EXG: exergame group; Post: after the intervention; Pre: before the intervention; RT: response time.

There was no significant group×time interaction in the Eriksen flanker test congruent and incongruent accuracy and RT. However, EXG had a significantly shortened congruent RT postintervention (mean 1602.6 ms, SD 433.8 ms, vs mean 1465.6 ms, SD 412.5 ms, *t*_12_=2.419, *P*=.03, 95% CI 13.581-260.419), while the RT for AEG did not change (mean 1454.6 ms, SD 550.0 ms, vs mean 1311.9 ms, SD 402.4 ms, *t*_12_=2.340, *P*=.31, 95% CI –151.794 to 437.067); see Figure 3A. Similarly, the incongruent RT was significantly shortened in EXG (mean 1675.4 ms, SD 511.3 ms, vs mean 1463.6 ms, SD 331.4 ms, *t*_10_=1.079, *P*=.04, 95% CI 14.621-408.917) but not in AEG (mean 1506.5 ms, SD 578.3 ms, vs mean 1275.6 ms, SD 407.7 ms, *t*_10_=1.525, *P*=.16, 95% CI –106.529 to 568.347); see Figure 3B.

### Event-Related Potential Data

#### N2 Component

**Figure 4:**
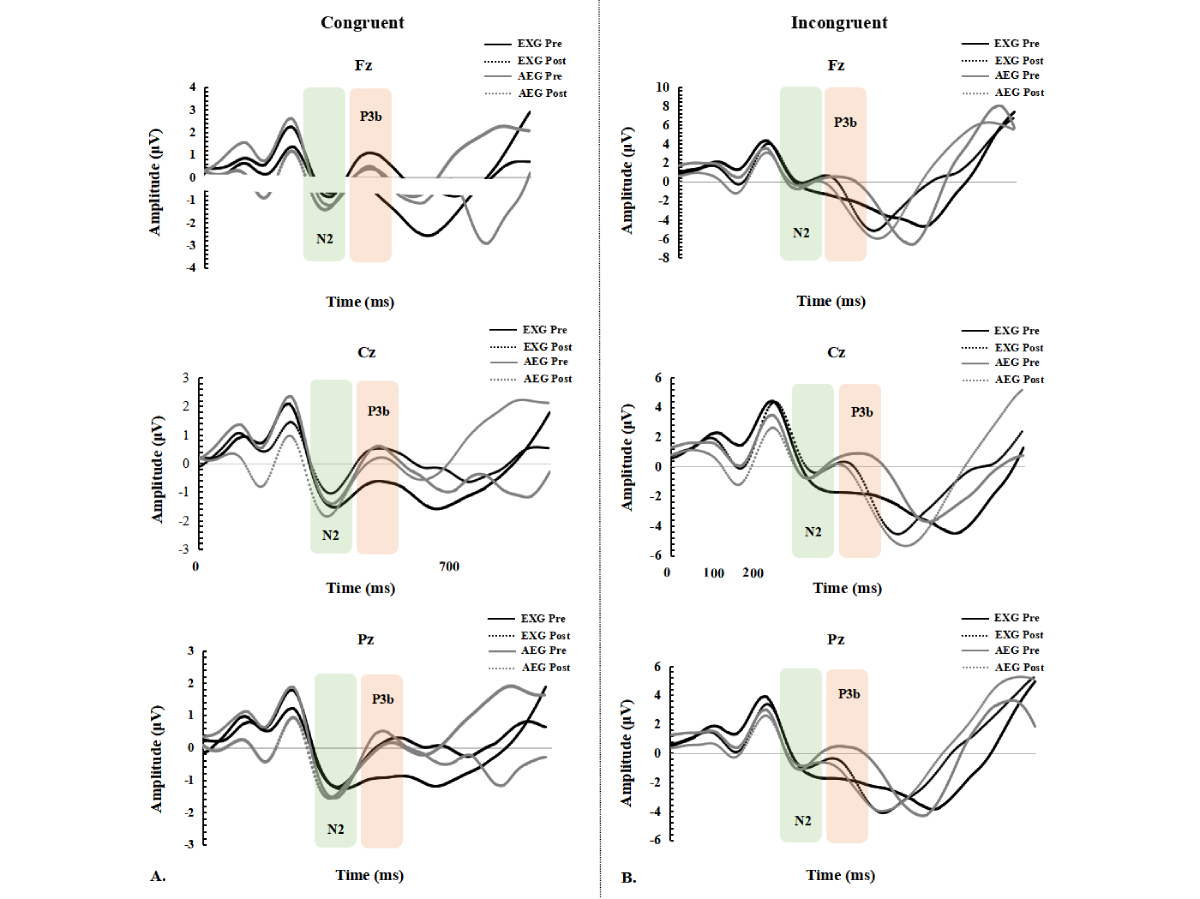
Average ERP waveforms of electrodes (Fz, Cz, Pz) for mean N2 and P3b amplitudes during the Eriksen flanker test. AEG: aerobic exercise group; Cz: central; ERP: event-related potential; EXG: exergame group; Fz: frontal; Post: after the intervention; Pre: before the intervention; Pz: parietal.

Tables 3 and 4 show congruent and incongruent N2 amplitude and latency data, respectively. Figure 4 shows the waveforms of congruent and incongruent N2 amplitudes pre- and postintervention. There was no significant group×time interaction in congruent and incongruent N2 amplitudes in Fz, Cz, and Pz cortices. However, EXG had a significantly increased congruent N2 amplitude in Fz, Cz, and Pz cortices postintervention, whereas AEG showed no significant changes. For the incongruent N2 amplitude in Fz, Cz, and Pz cortices, there were no significant changes in both EXG and AEG. However, there was a significant group×time interaction in congruent N2 latency in the Cz cortex but not in Fz and Pz cortices. EXG showed a significantly shortened congruent N2 latency in the Cz cortex postintervention, but AEG did not. Both EXG and AEG demonstrated no significant changes in congruent N2 latency in Fz and Pz cortices postintervention. For incongruent N2 latency in Fz, Cz, and Pz cortices, there was no significant group×time interaction. In addition, both EXG and AEG did not show a significant change in incongruent N2 latency in Fz, Cz, and Pz cortices postintervention.

**Table 3:** Comparison of Eriksen flanker test congruent N2/P3b component of EXG^a^ and AEG^b^.

Variable and effects		Group	Preintervention, mean (SD)	Postintervention, mean (SD)	*P* value^c,d^ (*η*^2^_p_)	95% CI	*P* value (*η*^2^_p_)
**N2 amplitude (μV)**							
	Fz^e^	EXG	–0.24 (1.15)	–0.97 (1.35)	0.016^c^ (0.582)	0.162 to 1.295	0.182 (0.079)
	Fz	AEG	–0.90 (1.59)	–0.91 (1.08)	0.990^c^ (0.007)	–1.067 to 1.080	0.182 (0.079)
	Cz^f^	EXG	–0.61 (1.27)	–1.24 (1.09)	0.027^c^ (0.532)	0.083 to 1.171	0.428 (0.029)
	Cz	AEG	–1.03 (1.18)	–1.23 (1.23)	0.693^c^ (0.166)	–0.900 to 1.302	0.428 (0.029)
	Pz^g^	EXG	–0.75 (1.21)	–1.24 (1.10)	0.045^c^ (0.424)	0.014 to 0.977	0.216 (0.069)
	Pz	AEG	–1.36 (1.20)	–1.15 (1.14)	0.708^c^ (0.179)	–1.420 to 1.001	0.216 (0.069)
**N2 latency (ms)**							
	Fz	EXG	319.2 (49.6)	290.3 (55.2)	0.176^d^ (0.551)	–15.259 to 73.059	0.207 (0.071)
	Fz	AEG	327.5 (36.4)	327.3 (36.3)	0.655^d^ (0.004)	–0.225 to 0.516	0.207 (0.071)
	Cz	EXG	336.0 (24.9)	303.5 (49.0)	0.044^d^ (0.836)	3.175 to 61.779	0.049 (0.163)
	Cz	AEG	336.0 (17.5)	335.6 (27.1)	0.655^d^ (0.015)	–11.990 to 12.633	0.049 (0.163)
	Pz	EXG	329.1 (33.5)	313.1 (42.2)	0.141^d^ (0.419)	–4.821 to 36.698	0.082 (0.131)
	Pz	AEG	331.4 (29.3)	335.6 (27.1)	0.317^d^ (0.151)	–13.764 to 5.236	0.082 (0.131)
**P3b amplitude (μV)**							
	Fz	EXG	1.06 (1.16)	2.40 (1.93)	0.033^d^ (0.842)	–2.525 to –0.173	0.018 (0.229)
	Fz	AEG	1.27 (1.19)	0.97 (0.94)	0.594^d^ (0.940)	–0.339 to 0.941	0.018 (0.229)
	Cz	EXG	0.68 (1.23)	1.88 (1.85)	0.044^c^ (0.764)	–2.375 to –0.391	0.023 (0.213)
	Cz	AEG	1.54 (1.81)	0.88 (0.98)	0.248^c^ (0.453)	–0.538 to 1.859	0.023 (0.213)
	Pz	EXG	0.52 (1.05)	1.25 (1.24)	0.084^c^ (0.870)	–1.586 to 0.116	0.049 (0.164)
	Pz	AEG	1.31 (1.55)	0.83 (1.02)	0.299^c^ (0.366)	–0.504 to 1.480	0.049 (0.164)
**P3b latency (ms)**							
	Fz	EXG	496.5 (82.2)	493.2 (72.4)	1.000^d^ (0.042)	–23.478 to 29.986	0.869 (0.001)
	Fz	AEG	488.7 (88.1)	487.8 (85.0)	0.833^d^ (0.010)	–11.088 to 12.852	0.869 (0.001)
	Cz	EXG	502.5 (83.7)	514.5 (80.1)	0.413^c^ (0.148)	–43.131 to 18.946	0.488 (0.022)
	Cz	AEG	503.0 (72.7)	502.3 (81.0)	0.947^c^ (0.009)	–22.002 to 23.402	0.488 (0.022)
	Pz	EXG	504.8 (105.0)	527.5 (85.8)	0.270^c^ (0.236)	–65.169 to 19.954	0.130 (0.101)
	Pz	AEG	507.5 (75.7)	492.3 (93.0)	0.238^c^ (0.179)	–11.800 to 42.200	0.130 (0.101)

^a^EXG: exergame group.

^b^AEG: aerobic exercise group.

^c^Paired *t* test *P* value.

^d^Wilcoxon signed-rank test *P* value.

^e^Fz: frontal.

^f^Cz: central.

^g^Pz: parietal.

**Table 4:** Comparison of Eriksen flanker test incongruent N2/P3b component of EXG^a^ and AEG^b^.

Variable and effects		Group	Preintervention, mean (SD)	Postintervention, mean (SD)	*P* value^c,d^ (*η*^2^_p_)	95% CI	*P* value (*η*^2^_p_)
**N2 amplitude (μV)**							
	Fz^e^	EXG	–0.18 (2.38)	–0.09 (2.36)	0.909^c^ (0.038)	–1.801 to 1.617	0.770 (0.004)
	Fz	AEG	–0.97 (2.43)	–0.60 (1.95)	0.401^c^ (0.168)	–1.308 to 0.568	0.770 (0.004)
	Cz^f^	EXG	–0.77 (2.30)	–0.22 (2.49)	0.533^c^ (0.229)	–2.409 to1.311	0.625 (0.011)
	Cz	AEG	–1.03 (1.79)	–1.00 (2.10)	0.960^c^ (0.015)	–1.208 to 1.153	0.625 (0.011)
	Pz^g^	EXG	–0.94 (2.16)	–0.86 (2.09)	0.921^c^ (0.038)	–1.763 to 1.605	0.845 (0.002)
	Pz	AEG	–1.42 (1.54)	–1.14 (1.92)	0.638^c^ (0.161)	–1.534 to 0.984	0.845 (0.002)
**N2 latency (ms)**							
	Fz	EXG	340.8 (10.8)	333.9 (25.3)	0.285^d^ (0.355)	–8.419 to 22.265	0.788 (0.003)
	Fz	AEG	332.8 (26.8)	322.8 (36.0)	0.465^d^ (0.313)	–9.500 to 29.390	0.788 (0.003)
	Cz	EXG	340.8 (10.8)	333.3 (25.5)	0.273^d^ (0.038)	–7.638 to 22.699	0.294 (0.050)
	Cz	AEG	330.3 (27.1)	331.7 (27.8)	0.715^d^ (0.161)	–9.743 to 6.907	0.294 (0.050)
	Pz	EXG	340.5 (11.9)	334.5 (24.7)	0.465^d^ (0.311)	–10.319 to 22.381	0.751 (0.005)
	Pz	AEG	332.4 (26.8)	329.2 (28.3)	0.285^d^ (0.116)	–4.827 to 11.208	0.751 (0.005)
**P3b amplitude (μV)**							
	Fz	EXG	0.92 (2.61)	2.88 (1.92)	0.002^c^ (1.215)	–3.386 to –0.635	0.009 (0.274)
	Fz	AEG	2.23 (2.59)	1.35 (1.84)	0.314^c^ (0.335)	–0.881 to 2.483	0.009 (0.274)
	Cz	EXG	0.48 (2.03)	2.88 (1.92)	0.002^c^ (1.215)	–3.681 to –1.121	0.001 (0.409)
	Cz	AEG	2.23 (2.59)	1.35 (1.84)	0.170^c^ (0.392)	–0.447 to 2.207	0.001 (0.409)
	Pz	EXG	0.10 (2.05)	2.21 (1.69)	0.003 (1.123)	–3.337 to –0.898	0.001 (0.385)
	Pz	AEG	1.72 (2.12)	1.10 (1.90)	0.201 (0.308)	–0.386 to 1.617	0.001 (0.385)
**P3b latency (ms)**							
	Fz	EXG	459.5 (86.0)	442.6 (85.6)	0.575^c^ (0.197)	–47.107 to 80.937	0.662 (0.009)
	Fz	AEG	465.2 (94.3)	432.1 (78.7)	0.108^c^ (0.380)	–8.696 to 74.733	0.662 (0.009)
	Cz	EXG	455.3 (86.4)	439.3 (94.7)	0.610^c^ (0.176)	–50.359 to 82.282	0.839 (0.002)
	Cz	AEG	476.2 (77.2)	453.1 (66.1)	0.064^c^ (0.321)	–1.600 to 47.800	0.839 (0.002)
	Pz	EXG	457.1 (85.0)	435.1 (100.1)	0.510^c^ (0.237)	–48.530 to 92.546	0.941 (0.000)
	Pz	AEG	477.6 (76.5)	452.8 (71.4)	0.053^c^ (0.335)	1.005 to 48.540	0.941 (0.000)

^a^EXG: exergame group.

^b^AEG: aerobic exercise group.

^c^Paired *t* test *P* value.

^d^Wilcoxon signed-rank test *P* value.

^e^Fz: frontal.

^f^Cz: central.

^g^Pz: parietal.

#### P3b Component

As shown in Tables 3 and 4, there were significant group×time interaction effects in both congruent and incongruent P3b amplitudes in Fz, Cz, and Pz cortices. EXG showed a significantly increased congruent P3b amplitude in Fz and Cz cortices postintervention but not in the Pz cortex. However, there was no significant change in the congruent P3b amplitude in Fz, Cz, and Pz cortices in AEG postintervention. Similarly, EXG showed a significantly increased incongruent P3b amplitude in Fz, Cz, and Pz cortices postintervention, but AEG did not. However, there were no significant group×time interaction effects in both congruent and incongruent P3b latency in Fz, Cz, and Pz cortices. In addition, both EXG and AEG did not show a significant change in incongruent P3b latency in Fz, Cz, and Pz cortices postintervention. The waveforms of congruent and incongruent P3b amplitudes in Fz, Cz, and Pz cortices for EXG and AEG pre- and postintervention are shown in Figure 4.

### Senior Fitness Test

**Figure 5:**
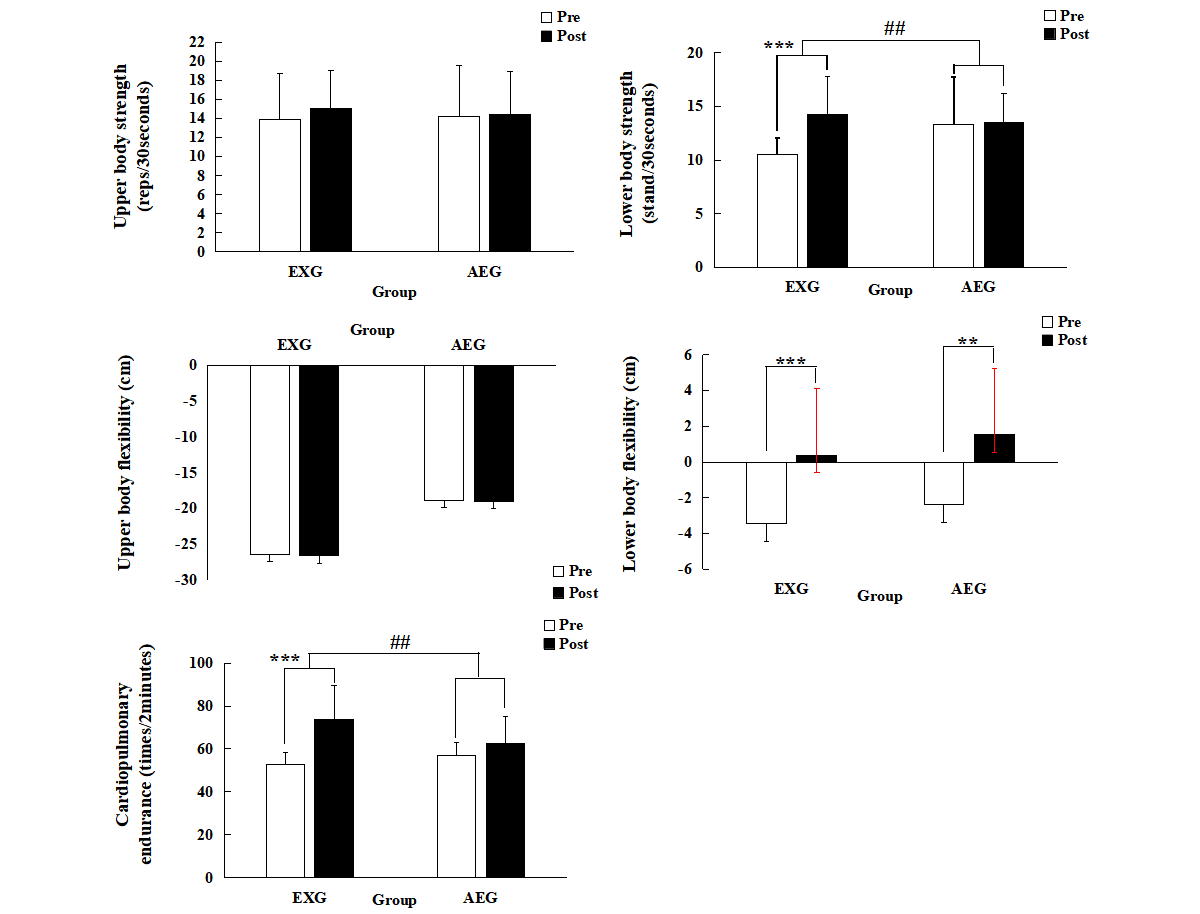
SFT before and after the 12-week exercise intervention. AEG: aerobic exercise group; EXG: exergame group; Post: after the intervention; Pre: before the intervention; SFT: senior fitness test.

The results of SFT in EXG and AEG postintervention are shown in Figure 5. There were significant group×time interaction effects on cardiopulmonary endurance (*F*_1.22_=7.434, *P*=.01, *η*^2^_p_=0.253). EXG showed a significantly increased number of 2-minute steps postintervention (mean 52.6, SD 5.5 times/2 minutes, vs mean 73.8, SD 15.8 times/2 minutes, *t*_12_=–4.791, *P*=.001, 95% CI –30.775 to –11.533), but AEG did not show a significantly change. Similarly, there were significant group×time interaction effects in lower body strength (*F*_1.22_=6.451, *P*=.02, *η*^2^_p_=0.227). EXG showed a significant increase in the number of full stands 30 seconds postintervention (mean 10.5, SD 1.6 stands/30 seconds, vs mean 14.2, SD 3.6 stands/30 seconds, *t*_12_=–4.688, *P*=.001, 95% CI –5.408 to –1.976), whereas AEG showed no significant change. For lower body flexibility, there was no significant group×time interaction, but EXG showed a significantly reduced distance between extended fingers and the toes postintervention (mean –3.5, SD 3.6 cm, vs mean 0.4, SD 3.7 cm, *t*_12_=–4.391, *P*=.01, 95% CI –5.755 to –1.938), and AEG also showed a significantly reduced distance (mean –2.4, SD 4.2 cm, vs mean 1.5, SD 3.7 cm, *t*_10_=–3.341, *P*=.01, 95% CI –6.364 to –1.272). For upper body strength and flexibility, the group×time interaction was not significant, and neither EXG nor AEG showed a significant change.

### Body Composition

**Figure 6:**
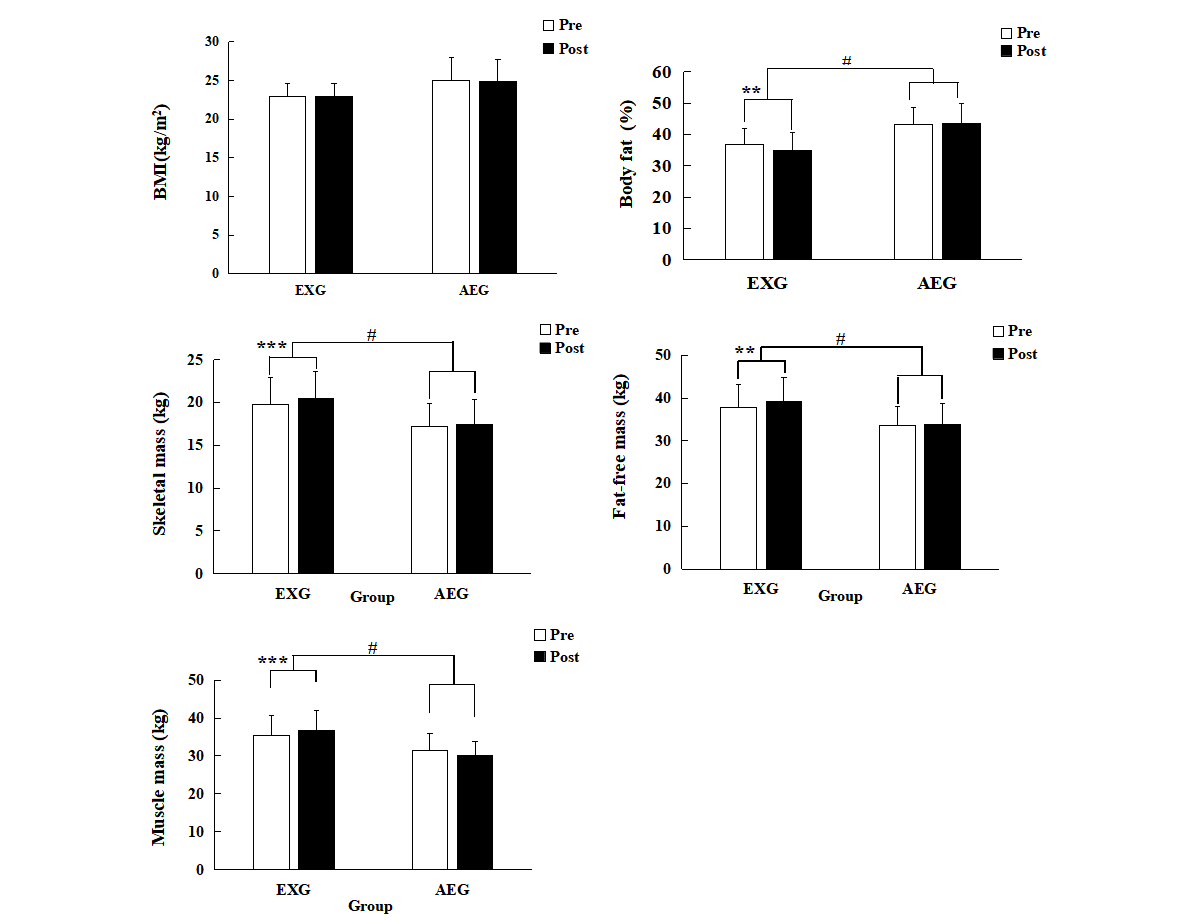
Body composition before and after the 12-week exercise intervention. AEG: aerobic exercise group; EXG: exergame group; Post: after the intervention; Pre: before the intervention.

Changes in body composition for EXG and AEG pre- and postintervention are shown in Figure 6. There was a significant group×time interaction in body fat percentage (*F*_1.22_=6.476, *P*=.02, *η*^2^_p_=0.236). EXG showed a significantly increased body fat percentage postintervention (mean 36.7.5%, SD 5.0%, vs mean 35.0%, SD 5.5%, *t*_11_=3.618, *P*=.004, 95% CI 0.702-2.882), whereas AEG showed no significant change. Similarly, there was a significant group×time interaction in skeletal mass (*F*_1.22_=4.525, *P*=.05, *η*^2^_p_=0.177). EXG showed significantly increased skeletal mass postintervention (mean 19.7, SD 3.2 kg, vs mean 20.5, SD 3.2 kg, *t*_11_=–5.297, *P*=.002, 95% CI –1.015 to –0.419), whereas AEG showed no significant change. There was a significant group×time interaction in fat-free mass (*F*_1.22_=6.103, *P*=.02, *η*^2^_p_=0.225). EXG showed significantly increased the fat-free mass postintervention (mean 37.7, SD 5.5 kg, vs mean 39.2, SD 5.7 kg, *t*_11_=–3.953, *P*=.002, 95% CI –2.296 to –0.654), whereas AEG showed no significant change. There was a significant group×time interaction in muscle mass (*F*_1.22_=6.636, *P*=.02, *η*^2^_p_=0.240). EXG showed significantly increased fat-free mass postintervention (mean 35.5, SD 5.3 kg, vs mean 36.6, SD 5.4 kg, *t*_11_=–4.712, *P*=.002, 95% CI –1.675 to –0.608), whereas AEG showed no significant change. For BMI, there was no significant group×time interaction, and neither EXG nor AEG showed a significant change.

## Discussion

### Principal Findings

This study investigated the effects of an exergame and aerobic cycling on executive and physical functions in older adults with dementia. We found a significantly shorter Ericksen flanker test congruent and incongruent RT in the response to exergame training. Moreover, exergaming generally yielded larger increases in neural activities that are related to attention and working memory and greater enhancement in lower body muscle strength and cardiorespiratory endurance compared to aerobic exercise.

A shorter Ericksen flanker test RT following exergame training in both congruent and incongruent signals in the study indicates the greater facilitation of executive function. In our previous findings, both exergaming and treadmill exercise resulted in a significantly shorter Stroop task RT in patients with metabolic syndrome through improvements in basic information processing and executive function suppression control [40]. Another study reported that combining physical and cognitive stimulation in 8 weeks of multimodal exercise substantially improves information processing speed in older adults [41]. Collectively, participating in aerobic exercise training improves information processing speed in older adults regardless of the exercise intensity and type. Consistent with the results of these previous studies, the results of this study showed that exergame training results in a significant improvement in information processing speed, suggesting that auditory, visual, and motor sensory stimulation during exergaming may accelerate information processing speed in older adults with dementia. Although there was no significant interaction between the group and the intervention in this study, there was no significant change in the Ericksen flanker test RT for AEG. Thus, we cannot rule out the possibility that exergaming has greater beneficial effects on information processing speed than general aerobic exercise, which should be further clarified in future longitudinal studies with a larger sample size.

In this study, exergame training significantly increased the congruent N2 amplitude in Fz and Cz cortices and reduced latency in the Cz cortex. Distinguishing stimulus and cognitive processes are closely related to attention during the tasks [42]. Hwang et al found reduced N2 activation in the central lobe and occipital cortex after acute aerobic exercise [43]. Similarly, a decreased N2 amplitude and longer N2 latency were observed during exercise [44]. We previously reported an increased N2 amplitude in response to exergame training, suggesting that exergaming effectively increases selective attention by regulating the activities of the central and occipital cortices [40]. Performing exercise in a virtual environment also increases the N2 amplitude and reduces latency [31]. These changes in brain activity indicate that exercise with extra-environmental stimulation may promote better decision-making (frontal and central areas) and visual perception (occipital areas). In this context, using cognition-stimulating games may facilitate the ability to clearly distinguish stimuli by activating the cerebral cortex with visual and auditory stimuli, thereby promoting cognitive processes. Therefore, our findings suggest that exergaming improves attention by effectively promoting neuronal activity in older individuals with dementia. Furthermore, considering that the N2 latency after exergaming was shorter than that after aerobic exercise, and the congruent N2 amplitude and latency did not change with aerobic exercise, it is still possible that exergaming may have a greater effect on neural activity related to executive function and attention compared to a simple form of aerobic exercise.

We also found a greater increase in congruent and incongruent P3b amplitudes after the exergame than after aerobic exercise in the study. This suggests that exergaming may yield greater effects on working memory than a simple mode of aerobic exercise. Tsai et al reported that performing resistance training for 12 months is associated with maintaining the capacities for allocating attention, as measured by the P3b amplitude in healthy older males [45]. Our previous investigation also found that aerobic exercise and resistance exercise have positive effects on neuronal activation, especially on the P3b amplitude in young adults [46]. Additionally, exergaming improves categorization of the incoming information and updating the context of working memory, as measured by the P300 amplitude in patients with metabolic syndrome [40]. Taken together, both exercise and exergaming induce brain activity through visual perceptual stimuli and activate nerve cells in the brain to promote information processing related to stimuli judgment and decision-making. Together, our findings elucidate that exergaming can be a more effective intervention in improving the classification of input information and working memory in the elderly with dementia compared to general aerobic exercise.

A recent meta-analysis showed that interventions combining both physical and cognitive training improve cognitive function than physical training alone [47], and our previous study supports the results of the meta-analysis [40]. In turn, better executive function is thought to be supported by healthy lifestyle interventions, including physical activity [48]. It is also important to note that exergaming showed greater improvements in lower body muscle strength and cardiorespiratory endurance compared to aerobic exercise. During the Alchemist Treasure game in the ExerHeart device used in this study, participants had to continuously walk or adjust their body position. These constant body movements throughout the game may also have resulted in improvements in their cardiorespiratory endurance. Our results are consistent with a previous study showing that the exergame program effectively improves cardiopulmonary endurance and leg muscle strength in healthy middle-aged and older adults [49]. In addition, lower body strength is closely related to cardiopulmonary endurance [50], and aerobic exercise improves repetitive muscle contraction and cardiopulmonary endurance and enhances everyday abilities, such as climbing stairs or sitting and standing up from a chair [51]. Therefore, exergaming may be an effective exercise for improving the lower body muscle strength and cardiorespiratory endurance of older adults.

### Strengths and Limitations

This study is the first to address the neuropsychological effects of exergaming in older adults with dementia. Meanwhile, the 12-week well-supervised exergame program effectively increased executive and physical functions in older adults with dementia. Therefore, our intervention protocol can be used in hospital settings or regional dementia centers to improve physical and cognitive functions in older individuals with dementia.

The study also had a few limitations. First, our study did not have a nonexercise control group, warranting some caution in the interpretation of the results. Nevertheless, pre-post test designs are commonly used to assess the effectiveness of intervention over time, and our results are consistent with other exercise intervention studies that have shown significant interventional effects in older adults with dementia. Thus, it is not likely that our results simply reflect the passage of time or other nonspecific intervention effects. Second, we had a relatively small number of participants (N=24) and homogeneous characteristics (eg, all Asians). As a result, the statistical power may be lower in this study, but at least to compensate for this problem, the effect sizes were calculated as references. Third, the MMSE score was measured only at baseline and was missing postintervention. Finally, since our study was limited to using only a single exergame, it is necessary to verify the effectiveness of other games. Therefore, these limitations may need to be supplemented in future studies.

### Conclusion

This study shows that exergaming is an effective approach to improve executive and physical functions in older adults with dementia. Therefore, regular participation in exergaming can be suggested as an effective alternative to aerobic exercise for treatment and early prevention of dementia in older adults. Future studies need to replicate our results using a nonexercise control group and a large number of participants.

## Appendix

Multimedia Appendix 1 Participants played the video game with Exerheart.

Multimedia Appendix 2 CONSORT-eHEALTH checklist (V 1.6.1).

## Abbreviations

AEG: aerobic exercise group
CERAD-K: Korean Consortium to Establish a Registry for Alzheimer’s Disease
Cz: central
EEG: electroencephalography
ERP: event-related potential
EXG: exergame group
Fz: frontal
HR: heart rate
HRR: heart rate reserved
K-ADL: Korean Activity of Daily Living
MMSE-K: Korean Mini Mental State Examination
Pz: parietal
RT: response time
SFT: senior fitness test
