# Supplementary material for: The Effects of Exergaming on Executive and Physical Functions in Older Adults With Dementia: Randomized Controlled Trial
Source: J Med Internet Res. 2023 Mar 7;25:e39993. doi: 10.2196/39993 (PMC10031442; doi:10.2196/39993)
Supplement: Multimedia Appendix 2 [file jmir_v25i1e39993_app2.pdf]

CONSORT-EHEALTH (V 1.6.1) - Submission/Publication Form

The CONSORT-EHEALTH checklist is intended for authors of randomized trials evaluating web-based and Internet-based applications/interventions, including mobile interventions, electronic games (incl multiplayer games), social media, certain telehealth applications, and other interactive and/or networked electronic applications. Some of the items (e.g. all subitems under item 5 - description of the intervention) may also be applicable for other study designs.

The goal of the CONSORT EHEALTH checklist and guideline is to be
a) a guide for reporting for authors of RCTs,
b) to form a basis for appraisal of an ehealth trial (in terms of validity)

CONSORT-EHEALTH items/subitems are MANDATORY reporting items for studies published in the Journal of Medical Internet Research and other journals / scientific societies endorsing the checklist.

Items numbered 1., 2., 3., 4a., 4b etc are original CONSORT or CONSORT-NPT (non-pharmacologic treatment) items.
Items with Roman numerals (i., ii, iii, iv etc.) are CONSORT-EHEALTH extensions/clarifications.

As the CONSORT-EHEALTH checklist is still considered in a formative stage, we would ask that you also RATE ON A SCALE OF 1-5 how important/useful you feel each item is FOR THE PURPOSE OF THE CHECKLIST and reporting guideline (optional).

Mandatory reporting items are marked with a red *.
In the textboxes, either copy & paste the relevant sections from your manuscript into this form - please include any quotes from your manuscript in QUOTATION MARKS,
or answer directly by providing additional information not in the manuscript, or elaborating on why the item was not relevant for this study.

YOUR ANSWERS WILL BE PUBLISHED AS A SUPPLEMENTARY FILE TO YOUR PUBLICATION IN JMIR AND ARE CONSIDERED PART OF YOUR PUBLICATION (IF ACCEPTED).
Please fill in these questions diligently. Information will not be copyedited, so please use proper spelling and grammar, use correct capitalization, and avoid abbreviations.

DO NOT FORGET TO SAVE AS PDF _AND_ CLICK THE SUBMIT BUTTON SO YOUR ANSWERS ARE IN OUR DATABASE !!!

Citation Suggestion (if you append the pdf as Appendix we suggest to cite this paper in the caption):
Eysenbach G, CONSORT-EHEALTH Group
CONSORT-EHEALTH: Improving and Standardizing Evaluation Reports of Web-based and Mobile Health Interventions
J Med Internet Res 2011;13(4):e126
URL: http://www.jmir.org/2011/4/e126/
doi: 10.2196/jmir.1923

PMID: 22209829

wushanshan521533@gmail.com （未分享） 切换帐号

草稿已保存

*必填

Your name *

First Last

Shanshan Wu

Primary Affiliation (short), City, Country *

University of Toronto, Toronto, Canada

School of Physical Education & Health, Wenzh

Your e-mail address *

abc@gmail.com

wss1229@wzu.edu.cn

Title of your manuscript *

Provide the (draft) title of your manuscript.

The effects Exergame on the executive function and physical function in older adults with dementia: Randomized Controlled Trial

Name of your App/Software/Intervention *

If there is a short and a long/alternate name, write the short name first and add the long name in brackets.

Alchemist's Treasure

Evaluated Version (if any)

e.g. "V1", "Release 2017-03-01", "Version 2.0.27913"

Version 1.4

Language(s) *

What language is the intervention/app in? If multiple languages are available, separate by comma (e.g. "English, French")

Korean

URL of your Intervention Website or App

e.g. a direct link to the mobile app on app in appstore (itunes, Google Play), or URL of the website. If the intervention is a DVD or hardware, you can also link to an Amazon page.

您的回答

URL of an image/screenshot (optional)

您的回答

Accessibility *

Can an enduser access the intervention presently?

- ○ access is free and open
- ○ access only for special usergroups, not open
- ○ access is open to everyone, but requires payment/subscription/in-app purchases
- ● app/intervention no longer accessible
- ○ 其他:

Primary Medical Indication/Disease/Condition *

e.g. "Stress", "Diabetes", or define the target group in brackets after the condition, e.g. "Autism (Parents of children with)", "Alzheimers (Informal Caregivers of)"

Metabolic Syndrome, Alzheimers , ADHD

Primary Outcomes measured in trial *

comma-separated list of primary outcomes reported in the trial

The “reaction time (RT)” and "electrophysiologi

Secondary/other outcomes

Are there any other outcomes the intervention is expected to affect?

您的回答

Recommended "Dose" *

What do the instructions for users say on how often the app should be used?

- (selected) Approximately Daily
- Approximately Weekly
- Approximately Monthly
- Approximately Yearly
- "as needed"
- 其他:

Approx. Percentage of Users (starters) still using the app as recommended after 3 months *

- (selected) unknown / not evaluated
- 0-10%
- 11-20%
- 21-30%
- 31-40%
- 41-50%
- 51-60%
- 61-70%
- 71%-80%
- 81-90%
- 91-100%
- 其他:

Overall, was the app/intervention effective? *

- (selected) yes: all primary outcomes were significantly better in intervention group vs control
- partly: SOME primary outcomes were significantly better in intervention group vs control
- no statistically significant difference between control and intervention
- potentially harmful: control was significantly better than intervention in one or more outcomes
- inconclusive: more research is needed
- 其他:

Article Preparation Status/Stage *

At which stage in your article preparation are you currently (at the time you fill in this form)

- not submitted yet - in early draft status
- not submitted yet - in late draft status, just before submission
- submitted to a journal but not reviewed yet
- submitted to a journal and after receiving initial reviewer comments
- (selected) submitted to a journal and accepted, but not published yet
- published
- 其他:

Journal *

If you already know where you will submit this paper (or if it is already submitted), please provide the journal name (if it is not JMIR, provide the journal name under "other")

- ○ not submitted yet / unclear where I will submit this
- ◉ Journal of Medical Internet Research (JMIR)
- ○ JMIR mHealth and UHealth
- ○ JMIR Serious Games
- ○ JMIR Mental Health
- ○ JMIR Public Health
- ○ JMIR Formative Research
- ○ Other JMIR sister journal
- ○ 其他:

Is this a full powered effectiveness trial or a pilot/feasibility trial? *

- ◉ Pilot/feasibility
- ○ Fully powered

Manuscript tracking number *

If this is a JMIR submission, please provide the manuscript tracking number under "other" (The ms tracking number can be found in the submission acknowledgement email, or when you login as author in JMIR. If the paper is already published in JMIR, then the ms tracking number is the four-digit number at the end of the DOI, to be found at the bottom of each published article in JMIR)

- ○ no ms number (yet) / not (yet) submitted to / published in JMIR
- ◉ 其他: JMIR ms#39993

TITLE AND ABSTRACT

1a) TITLE: Identification as a randomized trial in the title

1a) Does your paper address CONSORT item 1a? *

I.e does the title contain the phrase "Randomized Controlled Trial"? (if not, explain the reason under "other")

- (●) yes
- ( ) 其他:

1a-i) Identify the mode of delivery in the title

Identify the mode of delivery. Preferably use “web-based” and/or “mobile” and/or “electronic game” in the title. Avoid ambiguous terms like “online”, “virtual”, “interactive”. Use “Internet-based” only if Intervention includes non-web-based Internet components (e.g. email), use “computer-based” or “electronic” only if offline products are used. Use “virtual” only in the context of “virtual reality” (3-D worlds). Use “online” only in the context of “online support groups”. Complement or substitute product names with broader terms for the class of products (such as “mobile” or “smart phone” instead of “iphone”), especially if the application runs on different platforms.

	1	2	3	4	5	
subitem not at all important	( )	( )	( )	( )	(●)	essential

清除所选内容

Does your paper address subitem 1a-i? *

Copy and paste relevant sections from manuscript title (include quotes in quotation marks "like this" to indicate direct quotes from your manuscript), or elaborate on this item by providing additional information not in the ms, or briefly explain why the item is not applicable/relevant for your study

The effects Exergame on the executive function and physical function in older adults with dementia: Randomized Controlled Trial

1a-ii) Non-web-based components or important co-interventions in title

Mention non-web-based components or important co-interventions in title, if any (e.g., “with telephone support”).

	1	2	3	4	5	
subitem not at all important	◉	○	○	○	○	essential

清除所选内容

Does your paper address subitem 1a-ii?

Copy and paste relevant sections from manuscript title (include quotes in quotation marks "like this" to indicate direct quotes from your manuscript), or elaborate on this item by providing additional information not in the ms, or briefly explain why the item is not applicable/relevant for your study

您的回答

1a-iii) Primary condition or target group in the title

Mention primary condition or target group in the title, if any (e.g., “for children with Type I Diabetes”)
Example: A Web-based and Mobile Intervention with Telephone Support for Children with Type I Diabetes: Randomized Controlled Trial

	1	2	3	4	5	
subitem not at all important	○	○	○	○	◉	essential

清除所选内容

Does your paper address subitem 1a-iii? *

Copy and paste relevant sections from manuscript title (include quotes in quotation marks "like this" to indicate direct quotes from your manuscript), or elaborate on this item by providing additional information not in the ms, or briefly explain why the item is not applicable/relevant for your study

The effects Exergame on the executive function and physical function in older adults with dementia: Randomized Controlled Trial

1b) ABSTRACT: Structured summary of trial design, methods, results, and conclusions

NPT extension: Description of experimental treatment, comparator, care providers, centers, and blinding status.

1b-i) Key features/functionalities/components of the intervention and comparator in the METHODS section of the ABSTRACT

Mention key features/functionalities/components of the intervention and comparator in the abstract. If possible, also mention theories and principles used for designing the site. Keep in mind the needs of systematic reviewers and indexers by including important synonyms. (Note: Only report in the abstract what the main paper is reporting. If this information is missing from the main body of text, consider adding it)

	1	2	3	4	5	
subitem not at all important	○	○	○	○	●	essential

清除所选内容

Does your paper address subitem 1b-i? *

Copy and paste relevant sections from the manuscript abstract (include quotes in quotation marks "like this" to indicate direct quotes from your manuscript), or elaborate on this item by providing additional information not in the ms, or briefly explain why the item is not applicable/relevant for your study

Background: Despite increasing interest in the effects of exergame on cognitive function, little is known about its effects on older adults with dementia.
Objective: The purpose of the present study was to investigate the effects of exergame in comparison to regular aerobic exercise on executive function and physical function in older adults with dementia.
Methods: Twenty-four older adults with moderate dementia (80 ± 4.7 years old) participated in the study. Participants were randomized into either exergame group (EXG, n=13) or aerobic exercise group (AEG, n=11). The interventions lasted for 12 weeks and the Ericksen Flanker task was administered to assess executive function. The Flanker task performance accuracy (%) and response time (RT) and event-related potential (N2 and P3b) during the task were measured. Physical function was assessed using senior fitness test (SFT) before and after the intervention.

1b-ii) Level of human involvement in the METHODS section of the ABSTRACT

Clarify the level of human involvement in the abstract, e.g., use phrases like “fully automated” vs. “therapist/nurse/care provider/physician-assisted” (mention number and expertise of providers involved, if any). (Note: Only report in the abstract what the main paper is reporting. If this information is missing from the main body of text, consider adding it)

	1	2	3	4	5	
subitem not at all important	◉	○	○	○	○	essential

清除所选内容

Does your paper address subitem 1b-ii?

Copy and paste relevant sections from the manuscript abstract (include quotes in quotation marks "like this" to indicate direct quotes from your manuscript), or elaborate on this item by providing additional information not in the ms, or briefly explain why the item is not applicable/relevant for your study

您的回答

1b-iii) Open vs. closed, web-based (self-assessment) vs. face-to-face assessments in the METHODS section of the ABSTRACT

Mention how participants were recruited (online vs. offline), e.g., from an open access website or from a clinic or a closed online user group (closed usergroup trial), and clarify if this was a purely web-based trial, or there were face-to-face components (as part of the intervention or for assessment). Clearly say if outcomes were self-assessed through questionnaires (as common in web-based trials). Note: In traditional offline trials, an open trial (open-label trial) is a type of clinical trial in which both the researchers and participants know which treatment is being administered. To avoid confusion, use “blinded” or “unblinded” to indicated the level of blinding instead of “open”, as “open” in web-based trials usually refers to “open access” (i.e. participants can self-enrol). (Note: Only report in the abstract what the main paper is reporting. If this information is missing from the main body of text, consider adding it)

	1	2	3	4	5	
subitem not at all important	○	○	○	○	◉	essential

清除所选内容

Does your paper address subitem 1b-iii?

Copy and paste relevant sections from the manuscript abstract (include quotes in quotation marks "like this" to indicate direct quotes from your manuscript), or elaborate on this item by providing additional information not in the ms, or briefly explain why the item is not applicable/relevant for your study

Methods: Twenty-four older adults with moderate dementia (80 ± 4.7 years old) participated in the study. Participants were randomized into either exergame group (EXG, n=13) or aerobic exercise group (AEG, n=11). The interventions lasted for 12 weeks and the Ericksen Flanker task was administered to assess executive function. The Flanker task performance accuracy (%) and response time (RT) and event-related potential (N2 and P3b) during the task were measured. Physical function was assessed using senior fitness test (SFT) before and after the intervention.

1b-iv) RESULTS section in abstract must contain use data

Report number of participants enrolled/assessed in each group, the use/uptake of the intervention (e.g., attrition/adherence metrics, use over time, number of logins etc.), in addition to primary/secondary outcomes. (Note: Only report in the abstract what the main paper is reporting. If this information is missing from the main body of text, consider adding it)

	1	2	3	4	5	
subitem not at all important	○	○	○	○	◉	essential

清除所选内容

Does your paper address subitem 1b-iv?

Copy and paste relevant sections from the manuscript abstract (include quotes in quotation marks "like this" to indicate direct quotes from your manuscript), or elaborate on this item by providing additional information not in the ms, or briefly explain why the item is not applicable/relevant for your study

Results: EXG, compared to AEG, demonstrated greater improvements in the SFT. While there was a significantly shorter RT in EXG after intervention, no changes occurred in AEG. EXG showed significantly increased N2 amplitude after intervention compared to baseline, but there were no significant changes in AEG. EXG also yielded a shorter N2 latency for Cz cortices during both congruent and incongruent conditions compared to the AEG. Lastly, EXG presented significantly increased congruent/incongruent P3b amplitude and no changes were observed in AEG.

1b-v) CONCLUSIONS/DISCUSSION in abstract for negative trials

Conclusions/Discussions in abstract for negative trials: Discuss the primary outcome - if the trial is negative (primary outcome not changed), and the intervention was not used, discuss whether negative results are attributable to lack of uptake and discuss reasons. (Note: Only report in the abstract what the main paper is reporting. If this information is missing from the main body of text, consider adding it)

	1	2	3	4	5	
subitem not at all important	○	○	○	○	◉	essential

清除所选内容

Does your paper address subitem 1b-v?

Copy and paste relevant sections from the manuscript abstract (include quotes in quotation marks "like this" to indicate direct quotes from your manuscript), or elaborate on this item by providing additional information not in the ms, or briefly explain why the item is not applicable/relevant for your study

Conclusions: Our results suggest that exergame may be associated with greater improvements in brain neuronal activity and enhanced executive function task performance than regular aerobic exercise. Exergame characterized by both aerobic exercise and cognitive stimulation can be employed as an effective intervention to improve cognitive and physical function in older adults with dementia.

INTRODUCTION

2a) In INTRODUCTION: Scientific background and explanation of rationale

2a-i) Problem and the type of system/solution

Describe the problem and the type of system/solution that is object of the study: intended as stand-alone intervention vs. incorporated in broader health care program? Intended for a particular patient population? Goals of the intervention, e.g., being more cost-effective to other interventions, replace or complement other solutions? (Note: Details about the intervention are provided in “Methods” under 5)

	1	2	3	4	5	
subitem not at all important	○	○	○	○	◉	essential

清除所选内容

Does your paper address subitem 2a-i? *

Copy and paste relevant sections from the manuscript (include quotes in quotation marks "like this" to indicate direct quotes from your manuscript), or elaborate on this item by providing additional information not in the ms, or briefly explain why the item is not applicable/relevant for your study

Executive function is a family of at least three different functions that include the maintenance and execution of target tasks by suppressing interference factors caused by unnecessary information in the information processing process [1]. Execution function is not a single structure but can be divided into (1) core: consists of inhibition, renewal / working memory, conversion; (2) higher-level: consists of plan / solve problems, and depends heavily on the frontal lobes [2, 3]. Executive dysfunction often represents age-related cognitive decline and dementia, which are also associated with declining ability to independently maintain activities of daily living [4-6]. Indeed, dementia-related deficits in executive function cause fatal impairment in activities in daily living in older adults [7, 8].

2a-ii) Scientific background, rationale: What is known about the (type of) system

Scientific background, rationale: What is known about the (type of) system that is the object of the study (be sure to discuss the use of similar systems for other conditions/diagnoses, if appropiate), motivation for the study, i.e. what are the reasons for and what is the context for this specific study, from which stakeholder viewpoint is the study performed, potential impact of findings [2]. Briefly justify the choice of the comparator.

	1	2	3	4	5	
subitem not at all important	○	○	○	○	◉	essential

清除所选内容

Does your paper address subitem 2a-ii? *

Copy and paste relevant sections from the manuscript (include quotes in quotation marks "like this" to indicate direct quotes from your manuscript), or elaborate on this item by providing additional information not in the ms, or briefly explain why the item is not applicable/relevant for your study

Executive function is a family of at least three different functions that include the maintenance and execution of target tasks by suppressing interference factors caused by unnecessary information in the information processing process [1]. Execution function is not a single structure but can be divided into (1) core: consists of inhibition, renewal / working memory, conversion; (2) higher-level: consists of plan / solve problems, and depends heavily on the frontal lobes [2, 3]. Executive dysfunction often represents age-related cognitive decline and dementia, which are also associated with declining ability to independently maintain activities of daily living [4-6]. Indeed, dementia-related deficits in executive function cause fatal impairment in activities in daily living in older adults [7, 8].

2b) In INTRODUCTION: Specific objectives or hypotheses

Does your paper address CONSORT subitem 2b? *

Copy and paste relevant sections from the manuscript (include quotes in quotation marks "like this" to indicate direct quotes from your manuscript), or elaborate on this item by providing additional information not in the ms, or briefly explain why the item is not applicable/relevant for your study

The present study shows that exergame is an effective approach to improve the executive and physical functions in older adults with dementia. Therefore, regular participation in exergame can be suggested as an effective alternative to aerobic exercise for treatment and early prevention of older adults with dementia. Future studies need to replicate the present results using a non-exercise control group and large number of participants.

METHODS

3a) Description of trial design (such as parallel, factorial) including allocation ratio

Does your paper address CONSORT subitem 3a? *

Copy and paste relevant sections from the manuscript (include quotes in quotation marks "like this" to indicate direct quotes from your manuscript), or elaborate on this item by providing additional information not in the ms, or briefly explain why the item is not applicable/relevant for your study

All participants had 2 weeks of familiarization period before starting 12 weeks of both exergame and aerobic exercise training. The frequency of both EXG and AEG was 3 days/week. The intensity of both groups was 60-70% of heart rate reserved (HRR) and was gradually increased (i.e., 30 minutes for 1-2 weeks, 35 minutes for 3 weeks, 40 minutes for 4-5 weeks, 45 minutes for 6-7 weeks, 50 minutes for 8-12 weeks). The reaction time (RT) and electrophysiological signal from the frontal (Fz), central (Cz), and parietal (Pz) cortex were collected during a Eriksen flanker task twice, pre- and post-exercise training. Physical function was assessed using senior fitness test (SFT) before and after the intervention.

3b) Important changes to methods after trial commencement (such as eligibility criteria), with reasons

Does your paper address CONSORT subitem 3b? *

Copy and paste relevant sections from the manuscript (include quotes in quotation marks "like this" to indicate direct quotes from your manuscript), or elaborate on this item by providing additional information not in the ms, or briefly explain why the item is not applicable/relevant for your study

All participants had 2 weeks of familiarization period before starting 12 weeks of both exergame and aerobic exercise training. The frequency of both EXG and AEG was 3 days/week. The intensity of both groups was 60-70% of heart rate reserved (HRR) and was gradually increased (i.e., 30 minutes for 1-2 weeks, 35 minutes for 3 weeks, 40 minutes for 4-5 weeks, 45 minutes for 6-7 weeks, 50 minutes for 8-12 weeks). The reaction time (RT) and electrophysiological signal from the frontal (Fz), central (Cz), and parietal (Pz) cortex were collected during a Eriksen flanker task twice, pre- and post-exercise training. Physical function was assessed using senior fitness test (SFT) before and after the intervention.

3b-i) Bug fixes, Downtimes, Content Changes

Bug fixes, Downtimes, Content Changes: ehealth systems are often dynamic systems. A description of changes to methods therefore also includes important changes made on the intervention or comparator during the trial (e.g., major bug fixes or changes in the functionality or content) (5-iii) and other “unexpected events” that may have influenced study design such as staff changes, system failures/downtimes, etc. [2].

	1	2	3	4	5	
subitem not at all important	◉	○	○	○	○	essential

清除所选内容

Does your paper address subitem 3b-i?

Copy and paste relevant sections from the manuscript (include quotes in quotation marks "like this" to indicate direct quotes from your manuscript), or elaborate on this item by providing additional information not in the ms, or briefly explain why the item is not applicable/relevant for your study

您的回答

4a) Eligibility criteria for participants

Does your paper address CONSORT subitem 4a? *

Copy and paste relevant sections from the manuscript (include quotes in quotation marks "like this" to indicate direct quotes from your manuscript), or elaborate on this item by providing additional information not in the ms, or briefly explain why the item is not applicable/relevant for your study

We recruited 52 sedentary older male and female adults with mild or moderate dementia from daycare centers in Busan, South Korea. All participants were diagnosed with dementia by their primary care physician and had comorbidities such as diabetes and hypertension prior to participating in the study. Inclusion criteria were as follows: ≥ 65 years; Mini Mental State Examination–Korea (MMSE-K) score 15–23, an indication of mild-to-moderate dementia; diagnosis of probable dementia based on comprehensive neuropsychological testing (Consortium to Establish a Registry for Alzheimer's Disease [CERAD] test battery); ability to perform physical exercise and daily life (Korean Activity of Daily Living [K-ADL]).

4a-i) Computer / Internet literacy

Computer / Internet literacy is often an implicit “de facto” eligibility criterion - this should be explicitly clarified.

	1	2	3	4	5	
subitem not at all important	(●)	( )	( )	( )	( )	essential

清除所选内容

Does your paper address subitem 4a-i?

Copy and paste relevant sections from the manuscript (include quotes in quotation marks "like this" to indicate direct quotes from your manuscript), or elaborate on this item by providing additional information not in the ms, or briefly explain why the item is not applicable/relevant for your study

您的回答

4a-ii) Open vs. closed, web-based vs. face-to-face assessments:

Open vs. closed, web-based vs. face-to-face assessments: Mention how participants were recruited (online vs. offline), e.g., from an open access website or from a clinic, and clarify if this was a purely web-based trial, or there were face-to-face components (as part of the intervention or for assessment), i.e., to what degree got the study team to know the participant. In online-only trials, clarify if participants were quasi-anonymous and whether having multiple identities was possible or whether technical or logistical measures (e.g., cookies, email confirmation, phone calls) were used to detect/prevent these.

	1	2	3	4	5	
subitem not at all important	○	○	○	○	◉	essential

清除所选内容

Does your paper address subitem 4a-ii? *

Copy and paste relevant sections from the manuscript (include quotes in quotation marks "like this" to indicate direct quotes from your manuscript), or elaborate on this item by providing additional information not in the ms, or briefly explain why the item is not applicable/relevant for your study

All participants had 2 weeks of familiarization period before starting 12 weeks of both exergame and aerobic exercise training. The frequency of both EXG and AEG was 3 days/week. The intensity of both groups was 60-70% of heart rate reserved (HRR) and was gradually increased (i.e., 30 minutes for 1-2 weeks, 35 minutes for 3 weeks, 40 minutes for 4-5 weeks, 45 minutes for 6-7 weeks, 50 minutes for 8-12 weeks). The reaction time (RT) and electrophysiological signal from the frontal (Fz), central (Cz), and parietal (Pz) cortex were collected during a Eriksen flanker task twice, pre- and post-exercise training. Physical function was assessed using senior fitness test (SFT) before and after the intervention.

4a-iii) Information giving during recruitment

Information given during recruitment. Specify how participants were briefed for recruitment and in the informed consent procedures (e.g., publish the informed consent documentation as appendix, see also item X26), as this information may have an effect on user self-selection, user expectation and may also bias results.

	1	2	3	4	5	
subitem not at all important	○	○	○	○	◉	essential

清除所选内容

Does your paper address subitem 4a-iii?

Copy and paste relevant sections from the manuscript (include quotes in quotation marks "like this" to indicate direct quotes from your manuscript), or elaborate on this item by providing additional information not in the ms, or briefly explain why the item is not applicable/relevant for your study

All participants completed a written informed consent form approved by the Institutional Review Board of Pusan National University (PNU IRB/2018_59_HR).

4b) Settings and locations where the data were collected

Does your paper address CONSORT subitem 4b? *

Copy and paste relevant sections from the manuscript (include quotes in quotation marks "like this" to indicate direct quotes from your manuscript), or elaborate on this item by providing additional information not in the ms, or briefly explain why the item is not applicable/relevant for your study

The reaction time (RT) and electrophysiological signal from the frontal (Fz), central (Cz), and parietal (Pz) cortex were collected during a Eriksen flanker task twice, pre- and post-exercise training. Physical function was assessed using senior fitness test (SFT) before and after the intervention.

4b-i) Report if outcomes were (self-)assessed through online questionnaires

Clearly report if outcomes were (self-)assessed through online questionnaires (as common in web-based trials) or otherwise.

	1	2	3	4	5	
subitem not at all important	○	○	○	○	◉	essential

清除所选内容

Does your paper address subitem 4b-i? *

Copy and paste relevant sections from the manuscript (include quotes in quotation marks "like this" to indicate direct quotes from your manuscript), or elaborate on this item by providing additional information not in the ms, or briefly explain why the item is not applicable/relevant for your study

The reaction time (RT) and electrophysiological signal from the frontal (Fz), central (Cz), and parietal (Pz) cortex were collected during a Eriksen flanker task twice, pre- and post-exercise training. Physical function was assessed using senior fitness test (SFT) before and after the intervention.

4b-ii) Report how institutional affiliations are displayed

Report how institutional affiliations are displayed to potential participants [on ehealth media], as affiliations with prestigious hospitals or universities may affect volunteer rates, use, and reactions with regards to an intervention.(Not a required item – describe only if this may bias results)

	1	2	3	4	5	
subitem not at all important	◉	○	○	○	○	essential

清除所选内容

Does your paper address subitem 4b-ii?

Copy and paste relevant sections from the manuscript (include quotes in quotation marks "like this" to indicate direct quotes from your manuscript), or elaborate on this item by providing additional information not in the ms, or briefly explain why the item is not applicable/relevant for your study

您的回答

5) The interventions for each group with sufficient details to allow replication, including how and when they were actually administered

5-i) Mention names, credential, affiliations of the developers, sponsors, and owners

Mention names, credential, affiliations of the developers, sponsors, and owners [6] (if authors/evaluators are owners or developer of the software, this needs to be declared in a “Conflict of interest” section or mentioned elsewhere in the manuscript).

	1	2	3	4	5	
subitem not at all important	○	○	○	○	○	essential

Does your paper address subitem 5-i?

Copy and paste relevant sections from the manuscript (include quotes in quotation marks "like this" to indicate direct quotes from your manuscript), or elaborate on this item by providing additional information not in the ms, or briefly explain why the item is not applicable/relevant for your study

您的回答

5-ii) Describe the history/development process

Describe the history/development process of the application and previous formative evaluations (e.g., focus groups, usability testing), as these will have an impact on adoption/use rates and help with interpreting results.

	1	2	3	4	5	
subitem not at all important	○	○	○	○	○	essential

Does your paper address subitem 5-ii?

Copy and paste relevant sections from the manuscript (include quotes in quotation marks "like this" to indicate direct quotes from your manuscript), or elaborate on this item by providing additional information not in the ms, or briefly explain why the item is not applicable/relevant for your study

您的回答

5-iii) Revisions and updating

Revisions and updating. Clearly mention the date and/or version number of the application/intervention (and comparator, if applicable) evaluated, or describe whether the intervention underwent major changes during the evaluation process, or whether the development and/or content was “frozen” during the trial. Describe dynamic components such as news feeds or changing content which may have an impact on the replicability of the intervention (for unexpected events see item 3b).

	1	2	3	4	5	
subitem not at all important	○	○	○	○	○	essential

Does your paper address subitem 5-iii?

Copy and paste relevant sections from the manuscript (include quotes in quotation marks "like this" to indicate direct quotes from your manuscript), or elaborate on this item by providing additional information not in the ms, or briefly explain why the item is not applicable/relevant for your study

您的回答

5-iv) Quality assurance methods

Provide information on quality assurance methods to ensure accuracy and quality of information provided [1], if applicable.

	1	2	3	4	5	
subitem not at all important	○	○	○	○	○	essential

Does your paper address subitem 5-iv?

Copy and paste relevant sections from the manuscript (include quotes in quotation marks "like this" to indicate direct quotes from your manuscript), or elaborate on this item by providing additional information not in the ms, or briefly explain why the item is not applicable/relevant for your study

您的回答

5-v) Ensure replicability by publishing the source code, and/or providing screenshots/screen-capture video, and/or providing flowcharts of the algorithms used

Ensure replicability by publishing the source code, and/or providing screenshots/screen-capture video, and/or providing flowcharts of the algorithms used. Replicability (i.e., other researchers should in principle be able to replicate the study) is a hallmark of scientific reporting.

	1	2	3	4	5	
subitem not at all important	○	○	○	○	○	essential

Does your paper address subitem 5-v?

Copy and paste relevant sections from the manuscript (include quotes in quotation marks "like this" to indicate direct quotes from your manuscript), or elaborate on this item by providing additional information not in the ms, or briefly explain why the item is not applicable/relevant for your study

您的回答

5-vi) Digital preservation

Digital preservation: Provide the URL of the application, but as the intervention is likely to change or disappear over the course of the years; also make sure the intervention is archived (Internet Archive, webcitation.org, and/or publishing the source code or screenshots/videos alongside the article). As pages behind login screens cannot be archived, consider creating demo pages which are accessible without login.

	1	2	3	4	5	
subitem not at all important	○	○	○	○	○	essential

Does your paper address subitem 5-vi?

Copy and paste relevant sections from the manuscript (include quotes in quotation marks "like this" to indicate direct quotes from your manuscript), or elaborate on this item by providing additional information not in the ms, or briefly explain why the item is not applicable/relevant for your study

您的回答

5-vii) Access

Access: Describe how participants accessed the application, in what setting/context, if they had to pay (or were paid) or not, whether they had to be a member of specific group. If known, describe how participants obtained "access to the platform and Internet" [1]. To ensure access for editors/reviewers/readers, consider to provide a "backdoor" login account or demo mode for reviewers/readers to explore the application (also important for archiving purposes, see vi).

	1	2	3	4	5	
subitem not at all important	○	○	○	○	◉	essential

清除所选内容

Does your paper address subitem 5-vii? *

Copy and paste relevant sections from the manuscript (include quotes in quotation marks "like this" to indicate direct quotes from your manuscript), or elaborate on this item by providing additional information not in the ms, or briefly explain why the item is not applicable/relevant for your study

AEG performed exercise using commercial recumbent cycle 704 (EGOJIN, China). The initial bicycle resistance was 0 Kp and the exercise intensity was increased by 1 Kp every 5 minutes. Different resistance between male and female was used during the exercise to account for the sex-related difference in the cardiorespiratory fitness and lower-body strength (e.g., Male: 1 to 3 weeks up to 3 Kp, 4 to 12 weeks up to 4 Kp; Female: 1 to 3 weeks up to 2 Kp, 4 to 12 weeks up to 3 Kp).
EXG performed exercise using ExerHeart® devices (D&J Humancare, Busan, South Korea) that comes with a running/jumping mat [950(W) × 1300(D) × 1700(H)] (Fig. 2A). ExerHeart® is an intelligent exercise management service developed by medical experts and exercise experts. We used a game called "Alchemist's Treasure", which is a running game based on the Talesrunner IP and co-developed with ExerHeart® (Fig. 2B). During playing "Alchemist's Treasure", players run with the avatar, avoiding obstacles, and win items using the front, back, left, and right while running or jumping at speed on the mat.

5-viii) Mode of delivery, features/functionalities/components of the intervention and comparator, and the theoretical framework

Describe mode of delivery, features/functionalities/components of the intervention and comparator, and the theoretical framework [6] used to design them (instructional strategy [1], behaviour change techniques, persuasive features, etc., see e.g., [7, 8] for terminology). This includes an in-depth description of the content (including where it is coming from and who developed it) [1],” whether [and how] it is tailored to individual circumstances and allows users to track their progress and receive feedback” [6]. This also includes a description of communication delivery channels and – if computer-mediated communication is a component – whether communication was synchronous or asynchronous [6]. It also includes information on presentation strategies [1], including page design principles, average amount of text on pages, presence of hyperlinks to other resources, etc. [1].

	1	2	3	4	5	
subitem not at all important	○	○	○	○	○	essential

Does your paper address subitem 5-viii? *

Copy and paste relevant sections from the manuscript (include quotes in quotation marks "like this" to indicate direct quotes from your manuscript), or elaborate on this item by providing additional information not in the ms, or briefly explain why the item is not applicable/relevant for your study

The treasure of an alchemist : the mobile ¦tness game content playing tales runner with EXERHEART
1. Download ‘The treasure of an alchemist’ game from the Google App Store.
2. Choose the Game mode
- speed mode : Run as fast as possible.
- pattern mode : Run with a given speed.
- item mode : Run collecting items
3. Choose the level.
- level1 : There are 3 boss patterns
- level 2 : There are 4 boss patterns
- level3 : There are 5 boss patterns
4. Buy needed items with gold.
5. Set your Calory tapping Calory setting button.
6. Tapping the start button.
7. Choose your character.
8. Tap the Item buy or Character select button and start game.

5-ix) Describe use parameters

Describe use parameters (e.g., intended “doses” and optimal timing for use). Clarify what instructions or recommendations were given to the user, e.g., regarding timing, frequency, heaviness of use, if any, or was the intervention used ad libitum.

	1	2	3	4	5	
subitem not at all important	○	○	○	○	○	essential

Does your paper address subitem 5-ix?

Copy and paste relevant sections from the manuscript (include quotes in quotation marks "like this" to indicate direct quotes from your manuscript), or elaborate on this item by providing additional information not in the ms, or briefly explain why the item is not applicable/relevant for your study

您的回答

5-x) Clarify the level of human involvement

Clarify the level of human involvement (care providers or health professionals, also technical assistance) in the e-intervention or as co-intervention (detail number and expertise of professionals involved, if any, as well as “type of assistance offered, the timing and frequency of the support, how it is initiated, and the medium by which the assistance is delivered”. It may be necessary to distinguish between the level of human involvement required for the trial, and the level of human involvement required for a routine application outside of a RCT setting (discuss under item 21 – generalizability).

	1	2	3	4	5	
subitem not at all important	○	○	○	○	○	essential

Does your paper address subitem 5-x?

Copy and paste relevant sections from the manuscript (include quotes in quotation marks "like this" to indicate direct quotes from your manuscript), or elaborate on this item by providing additional information not in the ms, or briefly explain why the item is not applicable/relevant for your study

您的回答

5-xi) Report any prompts/reminders used

Report any prompts/reminders used: Clarify if there were prompts (letters, emails, phone calls, SMS) to use the application, what triggered them, frequency etc. It may be necessary to distinguish between the level of prompts/reminders required for the trial, and the level of prompts/reminders for a routine application outside of a RCT setting (discuss under item 21 – generalizability).

	1	2	3	4	5	
subitem not at all important	○	○	○	○	○	essential

Does your paper address subitem 5-xi? *

Copy and paste relevant sections from the manuscript (include quotes in quotation marks "like this" to indicate direct quotes from your manuscript), or elaborate on this item by providing additional information not in the ms, or briefly explain why the item is not applicable/relevant for your study

AEG performed exercise using commercial recumbent cycle 704 (EGOJIN, China). The initial bicycle resistance was 0 Kp and the exercise intensity was increased by 1 Kp every 5 minutes. Different resistance between male and female was used during the exercise to account for the sex-related difference in the cardiorespiratory fitness and lower-body strength (e.g., Male: 1 to 3 weeks up to 3 Kp, 4 to 12 weeks up to 4 Kp; Female: 1 to 3 weeks up to 2 Kp, 4 to 12 weeks up to 3 Kp).
EXG performed exercise using ExerHeart® devices (D&J Humancare, Busan, South Korea) that comes with a running/jumping mat [950(W) × 1300(D) × 1700(H)] (Fig. 2A). ExerHeart® is an intelligent exercise management service developed by medical experts and exercise experts. We used a game called "Alchemist's Treasure", which is a running game based on the Talesrunner IP and co-developed with ExerHeart® (Fig. 2B). During playing “Alchemist's Treasure”, players run with the avatar, avoiding obstacles, and win items using the front, back, left, and right while running or jumping at speed on the mat.

5-xii) Describe any co-interventions (incl. training/support)

Describe any co-interventions (incl. training/support): Clearly state any interventions that are provided in addition to the targeted eHealth intervention, as ehealth intervention may not be designed as stand-alone intervention. This includes training sessions and support [1]. It may be necessary to distinguish between the level of training required for the trial, and the level of training for a routine application outside of a RCT setting (discuss under item 21 – generalizability.

	1	2	3	4	5	
subitem not at all important	○	○	○	○	○	essential

Does your paper address subitem 5-xii? *

Copy and paste relevant sections from the manuscript (include quotes in quotation marks "like this" to indicate direct quotes from your manuscript), or elaborate on this item by providing additional information not in the ms, or briefly explain why the item is not applicable/relevant for your study

All participants had 2 weeks of familiarization period before starting 12 weeks of both exergame and aerobic exercise training. The frequency of both EXG and AEG was 3 days/week. The intensity of both groups was 60-70% of heart rate reserved (HRR) and was gradually increased (i.e., 30 minutes for 1-2 weeks, 35 minutes for 3 weeks, 40 minutes for 4-5 weeks, 45 minutes for 6-7 weeks, 50 minutes for 8-12 weeks)

6a) Completely defined pre-specified primary and secondary outcome measures, including how and when they were assessed

Does your paper address CONSORT subitem 6a? *

Copy and paste relevant sections from the manuscript (include quotes in quotation marks "like this" to indicate direct quotes from your manuscript), or elaborate on this item by providing additional information not in the ms, or briefly explain why the item is not applicable/relevant for your study

All participants had 2 weeks of familiarization period before starting 12 weeks of both exergame and aerobic exercise training. The frequency of both EXG and AEG was 3 days/week. The intensity of both groups was 60-70% of heart rate reserved (HRR) and was gradually increased (i.e., 30 minutes for 1-2 weeks, 35 minutes for 3 weeks, 40 minutes for 4-5 weeks, 45 minutes for 6-7 weeks, 50 minutes for 8-12 weeks). All participants' heart rates (HR) were measured during exercise using HR monitors (polar RS400sd, Madison Height, Michigan, USA). HRR was calculated by using the Karvonen formula [37].
AEG performed exercise using commercial recumbent cycle 704 (EGOJIN, China). The initial bicycle resistance was 0 Kp and the exercise intensity was increased by 1 Kp every 5 minutes. Different resistance between male and female was used during the exercise to account for the sex-related difference in the cardiorespiratory fitness and lower-body strength (e.g., Male: 1 to 3 weeks up to 3 Kp, 4 to 12 weeks up to 4 Kp; Female: 1 to 3 weeks up to 2 Kp, 4 to 12 weeks up to 3 Kp).
EXG performed exercise using ExerHeart® devices (D&J Humancare, Busan, South Korea) that comes with a running/jumping mat [950(W) × 1300(D) × 1700(H)] (Fig. 2A). ExerHeart® is an intelligent exercise management service developed by medical experts and exercise experts. We used a game called "Alchemist's Treasure", which is a running game based on the Talesrunner IP and co-developed with ExerHeart® (Fig. 2B). During playing "Alchemist's Treasure", players run with the avatar, avoiding obstacles, and win items using the front, back, left, and right while running or jumping at speed on the mat.

6a-i) Online questionnaires: describe if they were validated for online use and apply CHERRIES items to describe how the questionnaires were designed/deployed

If outcomes were obtained through online questionnaires, describe if they were validated for online use and apply CHERRIES items to describe how the questionnaires were designed/deployed [9].

	1	2	3	4	5	
subitem not at all important	○	○	○	○	○	essential

Does your paper address subitem 6a-i?

Copy and paste relevant sections from manuscript text

您的回答

6a-ii) Describe whether and how “use” (including intensity of use/dosage) was defined/measured/monitored

Describe whether and how “use” (including intensity of use/dosage) was defined/measured/monitored (logins, logfile analysis, etc.). Use/adoption metrics are important process outcomes that should be reported in any ehealth trial.

	1	2	3	4	5	
subitem not at all important	○	○	○	○	○	essential

Does your paper address subitem 6a-ii?

Copy and paste relevant sections from manuscript text

您的回答

6a-iii) Describe whether, how, and when qualitative feedback from participants was obtained

Describe whether, how, and when qualitative feedback from participants was obtained (e.g., through emails, feedback forms, interviews, focus groups).

	1	2	3	4	5	
subitem not at all important	○	○	○	○	○	essential

Does your paper address subitem 6a-iii?

Copy and paste relevant sections from manuscript text

您的回答

6b) Any changes to trial outcomes after the trial commenced, with reasons

Does your paper address CONSORT subitem 6b? *

Copy and paste relevant sections from the manuscript (include quotes in quotation marks "like this" to indicate direct quotes from your manuscript), or elaborate on this item by providing additional information not in the ms, or briefly explain why the item is not applicable/relevant for your study

changes were made to trial outcomes after trial commenced

7a) How sample size was determined

NPT: When applicable, details of whether and how the clustering by care provides or centers was addressed

7a-i) Describe whether and how expected attrition was taken into account when calculating the sample size

Describe whether and how expected attrition was taken into account when calculating the sample size.

	1	2	3	4	5	
subitem not at all important	○	○	○	○	○	essential

Does your paper address subitem 7a-i?

Copy and paste relevant sections from manuscript title (include quotes in quotation marks "like this" to indicate direct quotes from your manuscript), or elaborate on this item by providing additional information not in the ms, or briefly explain why the item is not applicable/relevant for your study

您的回答

7b) When applicable, explanation of any interim analyses and stopping guidelines

Does your paper address CONSORT subitem 7b? *

Copy and paste relevant sections from the manuscript (include quotes in quotation marks "like this" to indicate direct quotes from your manuscript), or elaborate on this item by providing additional information not in the ms, or briefly explain why the item is not applicable/relevant for your study

Not applicable explanation of any interim analyses and stopping guidelines

8a) Method used to generate the random allocation sequence

NPT: When applicable, how care providers were allocated to each trial group

Does your paper address CONSORT subitem 8a? *

Copy and paste relevant sections from the manuscript (include quotes in quotation marks "like this" to indicate direct quotes from your manuscript), or elaborate on this item by providing additional information not in the ms, or briefly explain why the item is not applicable/relevant for your study

It's simple randomization using a random number generator

8b) Type of randomisation; details of any restriction (such as blocking and block size)

Does your paper address CONSORT subitem 8b? *

Copy and paste relevant sections from the manuscript (include quotes in quotation marks "like this" to indicate direct quotes from your manuscript), or elaborate on this item by providing additional information not in the ms, or briefly explain why the item is not applicable/relevant for your study

It's a two-group (exergaming and aerobic exercise group) simple

9) Mechanism used to implement the random allocation sequence (such as sequentially numbered containers), describing any steps taken to conceal the sequence until interventions were assigned

Does your paper address CONSORT subitem 9? *

Copy and paste relevant sections from the manuscript (include quotes in quotation marks "like this" to indicate direct quotes from your manuscript), or elaborate on this item by providing additional information not in the ms, or briefly explain why the item is not applicable/relevant for your study

random allocation sequence generator was used

10) Who generated the random allocation sequence, who enrolled participants, and who assigned participants to interventions

Does your paper address CONSORT subitem 10? *

Copy and paste relevant sections from the manuscript (include quotes in quotation marks "like this" to indicate direct quotes from your manuscript), or elaborate on this item by providing additional information not in the ms, or briefly explain why the item is not applicable/relevant for your study

clinical staff in daycare centers in Busan, South Korea the baseline survey, enrolled the participant, used the random allocation sequence generator to randomize participants into either exergame and aerobic exercise group.

11a) If done, who was blinded after assignment to interventions (for example, participants, care providers, those assessing outcomes) and how

NPT: Whether or not administering co-interventions were blinded to group assignment

11a-i) Specify who was blinded, and who wasn’t

Specify who was blinded, and who wasn’t. Usually, in web-based trials it is not possible to blind the participants [1, 3] (this should be clearly acknowledged), but it may be possible to blind outcome assessors, those doing data analysis or those administering co-interventions (if any).

	1	2	3	4	5	
subitem not at all important	○	○	○	○	○	essential

Does your paper address subitem 11a-i? *

Copy and paste relevant sections from the manuscript (include quotes in quotation marks "like this" to indicate direct quotes from your manuscript), or elaborate on this item by providing additional information not in the ms, or briefly explain why the item is not applicable/relevant for your study

the participant was blinded, the clinical staff was not.

11a-ii) Discuss e.g., whether participants knew which intervention was the “intervention of interest” and which one was the “comparator”

Informed consent procedures (4a-ii) can create biases and certain expectations - discuss e.g., whether participants knew which intervention was the “intervention of interest” and which one was the “comparator”.

	1	2	3	4	5	
subitem not at all important	○	○	○	○	○	essential

Does your paper address subitem 11a-ii?

Copy and paste relevant sections from the manuscript (include quotes in quotation marks "like this" to indicate direct quotes from your manuscript), or elaborate on this item by providing additional information not in the ms, or briefly explain why the item is not applicable/relevant for your study

您的回答

11b) If relevant, description of the similarity of interventions

(this item is usually not relevant for ehealth trials as it refers to similarity of a placebo or sham intervention to a active medication/intervention)

Does your paper address CONSORT subitem 11b? *

Copy and paste relevant sections from the manuscript (include quotes in quotation marks "like this" to indicate direct quotes from your manuscript), or elaborate on this item by providing additional information not in the ms, or briefly explain why the item is not applicable/relevant for your study

this item is usually not relevant for ehealth trials as it refers to similarity of a placebo or sham intervention to a active medication/intervention

12a) Statistical methods used to compare groups for primary and secondary outcomes

NPT: When applicable, details of whether and how the clustering by care providers or centers was addressed

Does your paper address CONSORT subitem 12a? *

Copy and paste relevant sections from the manuscript (include quotes in quotation marks "like this" to indicate direct quotes from your manuscript), or elaborate on this item by providing additional information not in the ms, or briefly explain why the item is not applicable/relevant for your study

We used the Shapiro-Wilk test to assess whether the data were normally distributed. The behavioral (i.e., accuracy rate and reaction time) and event-related potential (i.e., N2, P3b amplitude) data were analyzed using repeated-measures ANOVA to determine group (EXG vs AEG) × time (before vs after intervention) interaction. We also used the paired t-test (or Wilcoxon signed rank test) to examine the changes of each dependent variable after the intervention within each group. Bonferroni post-hoc analyzes were performed when there was a significant difference. Partial eta squared ($\eta 2p$) was used to assess the effect size. The statistical significance was set at alpha = 0.05. All statistical tests were conducted using SPSS (v. 24.0).

12a-i) Imputation techniques to deal with attrition / missing values

Imputation techniques to deal with attrition / missing values: Not all participants will use the intervention/comparator as intended and attrition is typically high in ehealth trials. Specify how participants who did not use the application or dropped out from the trial were treated in the statistical analysis (a complete case analysis is strongly discouraged, and simple imputation techniques such as LOCF may also be problematic [4]).

	1	2	3	4	5	
subitem not at all important	○	○	○	○	○	essential

Does your paper address subitem 12a-i? *

Copy and paste relevant sections from the manuscript (include quotes in quotation marks "like this" to indicate direct quotes from your manuscript), or elaborate on this item by providing additional information not in the ms, or briefly explain why the item is not applicable/relevant for your study

We used the Shapiro-Wilk test to assess whether the data were normally distributed. The behavioral (i.e., accuracy rate and reaction time) and event-related potential (i.e., N2, P3b amplitude) data were analyzed using repeated-measures ANOVA to determine group (EXG vs AEG) × time (before vs after intervention) interaction. We also used the paired t-test (or Wilcoxon signed rank test) to examine the changes of each dependent variable after the intervention within each group. Bonferroni post-hoc analyzes were performed when there was a significant difference. Partial eta squared (η2p) was used to assess the effect size. The statistical significance was set at alpha = 0.05. All statistical tests were conducted using SPSS (v. 24.0).

12b) Methods for additional analyses, such as subgroup analyses and adjusted analyses

Does your paper address CONSORT subitem 12b? *

Copy and paste relevant sections from the manuscript (include quotes in quotation marks "like this" to indicate direct quotes from your manuscript), or elaborate on this item by providing additional information not in the ms, or briefly explain why the item is not applicable/relevant for your study

There was no significant group × time interaction in the flanker congruent and incongruent accuracy and RT. However, EXG significantly shortened congruent RT after intervention (1602.6±433.8 ms vs. 1465.6±412.5 ms, p=0.032 η2p=0.324), while AEG did not change (1454.6±550.0 ms vs. 1311.9±402.4 ms, p=0.306 η2p=0.296) (Fig. 3A). Similarly, incongruent RT was significantly shortened in EXG (1675.4±511.3 ms vs. 1463.6±331.4 ms, p=0.037 η2p=0.492), but not in AEG (1506.5±578.3 ms vs. 1275.6±407.7 ms, p=0.158 η2p=0.462) (Fig. 3B).
Table 2 shows congruent and incongruent N2 amplitude and latency and Figure 4 shows the waveforms of congruent and incongruent N2 amplitude before and after intervention. There was no significant group × time interaction in congruent and incongruent N2 amplitude on Fz, Cz, and Pz. However, EXG significantly increased congruent N2 amplitude on Fz, Cz, and Pz after intervention, whereas AEG showed no significant changes. For incongruent N2 amplitude on Fz, Cz, and Pz, there were no significant changes in both EXG and AEG. On the other hand, there was a significant group × time interaction in congruent N2 latency on Cz, but not on Fz and Pz. EXG significantly shortened congruent N2 latency on Cz after intervention, but AEG did not. Both EXG and AEG did not significantly change congruent N2 latency on Fz and Pz after intervention. For incongruent N2 latency on Fz, Cz, and Pz, there was no significant group × time interaction. Also, both EXG and AEG did not significantly change incongruent N2 latency on Fz, Cz, and Pz after intervention.
As shown in Table 2, there were significant group × time interactions in both congruent and incongruent P3b amplitude on Fz, Cz, and Pz. EXG significantly increased congruent P3b amplitude on Fz and Cz after intervention, but not on Pz. However, AEG did not significantly change congruent P3b amplitude on Fz, Cz, and Pz after intervention. Similarly, EXG significantly increased incongruent P3b amplitude on Fz, Cz, and Pz after intervention, but AEG did not. On the other hand, there was no significant group × time interaction in both congruent and incongruent P3b latency on Fz, Cz, and Pz. In addition, both EXG and AEG did not significantly change incongruent P3b latency on Fz, Cz, and Pz after intervention. The waveforms of congruent and incongruent P3b amplitude Fz, Cz, and Pz for EXG and AEG before and after exercise are shown in Figure 4.
The results of SFT in EXG and AEG after intervention were shown in Figure 5. There was a significant group × time interaction in cardiopulmonary endurance (p=0.012, η2p=0.253). EXG significantly increased the number of 2-minute steps after intervention (52.6±5.5 times/2min vs. 73.8±15.8 times/2min, p=0.001, η2p=1.788), but AEG did not significantly change. Similarly, there was a significant group × time interaction in lower body strength (p=0.019, η2p=0.227). EXG significantly increased the number of full stands in 30 seconds after intervention (10.5±1.6 stand/30sec vs. 14.2±3.6 stand/30sec, p=0.001, η2p=1.327), whereas AEG showed no significant change. For lower body flexibility, there was no significant group × time interaction, but EXG significantly reduced the distance between extended fingers and tip of the toes after intervention (-3.5±3.6 cm vs. 0.4±3.7 cm, p=0.005, η2p=1.048) and AEG also significantly reduced (-2.4±4.2 cm vs. 1.6±3.7 cm, p=0.012, η2p=0.984). For upper body strength and flexibility, the group × time interaction was not significant, and neither EXG nor AEG were significantly changed.

X26) REB/IRB Approval and Ethical Considerations [recommended as subheading under "Methods"] (not a CONSORT item)

X26-i) Comment on ethics committee approval

	1	2	3	4	5	
subitem not at all important	○	○	○	○	○	essential

Does your paper address subitem X26-i?

Copy and paste relevant sections from the manuscript (include quotes in quotation marks "like this" to indicate direct quotes from your manuscript), or elaborate on this item by providing additional information not in the ms, or briefly explain why the item is not applicable/relevant for your study

您的回答

x26-ii) Outline informed consent procedures

Outline informed consent procedures e.g., if consent was obtained offline or online (how? Checkbox, etc.?), and what information was provided (see 4a-ii). See [6] for some items to be included in informed consent documents.

	1	2	3	4	5	
subitem not at all important	○	○	○	○	○	essential

Does your paper address subitem X26-ii?

Copy and paste relevant sections from the manuscript (include quotes in quotation marks "like this" to indicate direct quotes from your manuscript), or elaborate on this item by providing additional information not in the ms, or briefly explain why the item is not applicable/relevant for your study

您的回答

X26-iii) Safety and security procedures

Safety and security procedures, incl. privacy considerations, and any steps taken to reduce the likelihood or detection of harm (e.g., education and training, availability of a hotline)

	1	2	3	4	5	
subitem not at all important	○	○	○	○	○	essential

Does your paper address subitem X26-iii?

Copy and paste relevant sections from the manuscript (include quotes in quotation marks "like this" to indicate direct quotes from your manuscript), or elaborate on this item by providing additional information not in the ms, or briefly explain why the item is not applicable/relevant for your study

您的回答

RESULTS

13a) For each group, the numbers of participants who were randomly assigned, received intended treatment, and were analysed for the primary outcome

NPT: The number of care providers or centers performing the intervention in each group and the number of patients treated by each care provider in each center

Does your paper address CONSORT subitem 13a? *

Copy and paste relevant sections from the manuscript (include quotes in quotation marks "like this" to indicate direct quotes from your manuscript), or elaborate on this item by providing additional information not in the ms, or briefly explain why the item is not applicable/relevant for your study

52 participants were randomly assigned into two groups: exergame group (EXG, n=26) and aerobic exercise group (AEG, n=26).

13b) For each group, losses and exclusions after randomisation, together with reasons

Does your paper address CONSORT subitem 13b? (NOTE: Preferably, this is shown in a CONSORT flow diagram) *

Copy and paste relevant sections from the manuscript (include quotes in quotation marks "like this" to indicate direct quotes from your manuscript), or elaborate on this item by providing additional information not in the ms, or briefly explain why the item is not applicable/relevant for your study

No losses and exclusions after randomisation

13b-i) Attrition diagram

Strongly recommended: An attrition diagram (e.g., proportion of participants still logging in or using the intervention/comparator in each group plotted over time, similar to a survival curve) or other figures or tables demonstrating usage/dose/engagement.

	1	2	3	4	5	
subitem not at all important	○	○	○	○	○	essential

Does your paper address subitem 13b-i?

Copy and paste relevant sections from the manuscript or cite the figure number if applicable (include quotes in quotation marks "like this" to indicate direct quotes from your manuscript), or elaborate on this item by providing additional information not in the ms, or briefly explain why the item is not applicable/relevant for your study

您的回答

14a) Dates defining the periods of recruitment and follow-up

Does your paper address CONSORT subitem 14a? *

Copy and paste relevant sections from the manuscript (include quotes in quotation marks "like this" to indicate direct quotes from your manuscript), or elaborate on this item by providing additional information not in the ms, or briefly explain why the item is not applicable/relevant for your study

Participants were recruited from the daycare centers in Busan, South Korea， between March 1, 2018, and September 31, 2019.

14a-i) Indicate if critical “secular events” fell into the study period

Indicate if critical “secular events” fell into the study period, e.g., significant changes in Internet resources available or “changes in computer hardware or Internet delivery resources”

	1	2	3	4	5	
subitem not at all important	○	○	○	○	○	essential

Does your paper address subitem 14a-i?

Copy and paste relevant sections from the manuscript (include quotes in quotation marks "like this" to indicate direct quotes from your manuscript), or elaborate on this item by providing additional information not in the ms, or briefly explain why the item is not applicable/relevant for your study

您的回答

14b) Why the trial ended or was stopped (early)

Does your paper address CONSORT subitem 14b? *

Copy and paste relevant sections from the manuscript (include quotes in quotation marks "like this" to indicate direct quotes from your manuscript), or elaborate on this item by providing additional information not in the ms, or briefly explain why the item is not applicable/relevant for your study

Not trial ended or was stopped (early)

15) A table showing baseline demographic and clinical characteristics for each group

NPT: When applicable, a description of care providers (case volume, qualification, expertise, etc.) and centers (volume) in each group

Does your paper address CONSORT subitem 15? *

Copy and paste relevant sections from the manuscript (include quotes in quotation marks "like this" to indicate direct quotes from your manuscript), or elaborate on this item by providing additional information not in the ms, or briefly explain why the item is not applicable/relevant for your study

Table 1 showing baseline demographic and clinical characteristics for each group

15-i) Report demographics associated with digital divide issues

In ehealth trials it is particularly important to report demographics associated with digital divide issues, such as age, education, gender, social-economic status, computer/Internet/ehealth literacy of the participants, if known.

	1	2	3	4	5	
subitem not at all important	○	○	○	○	○	essential

Does your paper address subitem 15-i? *

Copy and paste relevant sections from the manuscript (include quotes in quotation marks "like this" to indicate direct quotes from your manuscript), or elaborate on this item by providing additional information not in the ms, or briefly explain why the item is not applicable/relevant for your study

Demographic and physical characteristics for all subjects are provided in Table1. There were no signi¦cant group differences at baseline measurements.

16) For each group, number of participants (denominator) included in each analysis and whether the analysis was by original assigned groups

16-i) Report multiple “denominators” and provide definitions

Report multiple “denominators” and provide definitions: Report N’s (and effect sizes) “across a range of study participation [and use] thresholds” [1], e.g., N exposed, N consented, N used more than x times, N used more than y weeks, N participants “used” the intervention/comparator at specific pre-defined time points of interest (in absolute and relative numbers per group). Always clearly define “use” of the intervention.

	1	2	3	4	5	
subitem not at all important	○	○	○	○	○	essential

Does your paper address subitem 16-i? *

Copy and paste relevant sections from the manuscript (include quotes in quotation marks "like this" to indicate direct quotes from your manuscript), or elaborate on this item by providing additional information not in the ms, or briefly explain why the item is not applicable/relevant for your study

the analysis was by original assigned groups

16-ii) Primary analysis should be intent-to-treat

Primary analysis should be intent-to-treat, secondary analyses could include comparing only “users”, with the appropriate caveats that this is no longer a randomized sample (see 18-i).

	1	2	3	4	5	
subitem not at all important	○	○	○	○	○	essential

Does your paper address subitem 16-ii?

Copy and paste relevant sections from the manuscript (include quotes in quotation marks "like this" to indicate direct quotes from your manuscript), or elaborate on this item by providing additional information not in the ms, or briefly explain why the item is not applicable/relevant for your study

您的回答

17a) For each primary and secondary outcome, results for each group, and the estimated effect size and its precision (such as 95% confidence interval)

Does your paper address CONSORT subitem 17a? *

Copy and paste relevant sections from the manuscript (include quotes in quotation marks "like this" to indicate direct quotes from your manuscript), or elaborate on this item by providing additional information not in the ms, or briefly explain why the item is not applicable/relevant for your study

Table 2 for each primary and secondary outcome, results for each group, and the estimated effect size and its precision

17a-i) Presentation of process outcomes such as metrics of use and intensity of use

In addition to primary/secondary (clinical) outcomes, the presentation of process outcomes such as metrics of use and intensity of use (dose, exposure) and their operational definitions is critical. This does not only refer to metrics of attrition (13-b) (often a binary variable), but also to more continuous exposure metrics such as "average session length". These must be accompanied by a technical description how a metric like a "session" is defined (e.g., timeout after idle time) [1] (report under item 6a).

	1	2	3	4	5	
subitem not at all important	○	○	○	○	○	essential

Does your paper address subitem 17a-i?

Copy and paste relevant sections from the manuscript (include quotes in quotation marks "like this" to indicate direct quotes from your manuscript), or elaborate on this item by providing additional information not in the ms, or briefly explain why the item is not applicable/relevant for your study

您的回答

17b) For binary outcomes, presentation of both absolute and relative effect sizes is recommended

Does your paper address CONSORT subitem 17b? *

Copy and paste relevant sections from the manuscript (include quotes in quotation marks "like this" to indicate direct quotes from your manuscript), or elaborate on this item by providing additional information not in the ms, or briefly explain why the item is not applicable/relevant for your study

Table 2 for binary outcomes, presentation of both absolute and relative effect sizes is recommended

18) Results of any other analyses performed, including subgroup analyses and adjusted analyses, distinguishing pre-specified from exploratory

Does your paper address CONSORT subitem 18? *

Copy and paste relevant sections from the manuscript (include quotes in quotation marks "like this" to indicate direct quotes from your manuscript), or elaborate on this item by providing additional information not in the ms, or briefly explain why the item is not applicable/relevant for your study

There was no significant group × time interaction in the flanker congruent and incongruent accuracy and RT. However, EXG significantly shortened congruent RT after intervention (1602.6±433.8 ms vs. 1465.6±412.5 ms, p=0.032 η2p=0.324), while AEG did not change (1454.6±550.0 ms vs. 1311.9±402.4 ms, p=0.306 η2p=0.296) (Fig. 3A). Similarly, incongruent RT was significantly shortened in EXG (1675.4±511.3 ms vs. 1463.6±331.4 ms, p=0.037 η2p=0.492), but not in AEG (1506.5±578.3 ms vs. 1275.6±407.7 ms, p=0.158 η2p=0.462) (Fig. 3B).
Table 2 shows congruent and incongruent N2 amplitude and latency and Figure 4 shows the waveforms of congruent and incongruent N2 amplitude before and after intervention. There was no significant group × time interaction in congruent and incongruent N2 amplitude on Fz, Cz, and Pz. However, EXG significantly increased congruent N2 amplitude on Fz, Cz, and Pz after intervention, whereas AEG showed no significant changes. For incongruent N2 amplitude on Fz, Cz, and Pz, there were no significant changes in both EXG and AEG. On the other hand, there was a significant group × time interaction in congruent N2 latency on Cz, but not on Fz and Pz. EXG significantly shortened congruent N2 latency on Cz after intervention, but AEG did not. Both EXG and AEG did not significantly change congruent N2 latency on Fz and Pz after intervention. For incongruent N2 latency on Fz, Cz, and Pz, there was no significant group × time interaction. Also, both EXG and AEG did not significantly change incongruent N2 latency on Fz, Cz, and Pz after intervention.
As shown in Table 2, there were significant group × time interactions in both congruent and incongruent P3b amplitude on Fz, Cz, and Pz. EXG significantly increased congruent P3b amplitude on Fz and Cz after intervention, but not on Pz. However, AEG did not significantly change congruent P3b amplitude on Fz, Cz, and Pz after intervention. Similarly, EXG significantly increased incongruent P3b amplitude on Fz, Cz, and Pz after intervention, but AEG did not. On the other hand, there was no significant group × time interaction in both congruent and incongruent P3b latency on Fz, Cz, and Pz. In addition, both EXG and AEG did not significantly change incongruent P3b latency on Fz, Cz, and Pz after intervention. The waveforms of congruent and incongruent P3b amplitude Fz, Cz, and Pz for EXG and AEG before and after exercise are shown in Figure 4.
The results of SFT in EXG and AEG after intervention were shown in Figure 5. There was a significant group × time interaction in cardiopulmonary endurance (p=0.012, η2p=0.253). EXG significantly increased the number of 2-minute steps after intervention (52.6±5.5 times/2min vs. 73.8±15.8 times/2min, p=0.001, η2p=1.788), but AEG did not significantly change. Similarly, there was a significant group × time interaction in lower body strength (p=0.019, η2p=0.227). EXG significantly increased the number of full stands in 30 seconds after intervention (10.5±1.6 stand/30sec vs. 14.2±3.6 stand/30sec, p=0.001, η2p=1.327), whereas AEG showed no significant change. For lower body flexibility, there was no significant group × time interaction, but EXG significantly reduced the distance between extended fingers and tip of the toes after intervention (-3.5±3.6 cm vs. 0.4±3.7 cm, p=0.005, η2p=1.048) and AEG also significantly reduced (-2.4±4.2 cm vs. 1.6±3.7 cm, p=0.012, η2p=0.984). For upper body strength and flexibility, the group × time interaction was not significant, and neither EXG nor AEG were significantly changed.

18-i) Subgroup analysis of comparing only users

A subgroup analysis of comparing only users is not uncommon in ehealth trials, but if done, it must be stressed that this is a self-selected sample and no longer an unbiased sample from a randomized trial (see 16-iii).

	1	2	3	4	5	
subitem not at all important	○	○	○	○	○	essential

Does your paper address subitem 18-i?

Copy and paste relevant sections from the manuscript (include quotes in quotation marks "like this" to indicate direct quotes from your manuscript), or elaborate on this item by providing additional information not in the ms, or briefly explain why the item is not applicable/relevant for your study

您的回答

19) All important harms or unintended effects in each group

(for specific guidance see CONSORT for harms)

Does your paper address CONSORT subitem 19? *

Copy and paste relevant sections from the manuscript (include quotes in quotation marks "like this" to indicate direct quotes from your manuscript), or elaborate on this item by providing additional information not in the ms, or briefly explain why the item is not applicable/relevant for your study

no unintended harms reported

19-i) Include privacy breaches, technical problems

Include privacy breaches, technical problems. This does not only include physical “harm” to participants, but also incidents such as perceived or real privacy breaches [1], technical problems, and other unexpected/unintended incidents. “Unintended effects” also includes unintended positive effects [2].

	1	2	3	4	5	
subitem not at all important	○	○	○	○	○	essential

Does your paper address subitem 19-i?

Copy and paste relevant sections from the manuscript (include quotes in quotation marks "like this" to indicate direct quotes from your manuscript), or elaborate on this item by providing additional information not in the ms, or briefly explain why the item is not applicable/relevant for your study

您的回答

19-ii) Include qualitative feedback from participants or observations from staff/researchers

Include qualitative feedback from participants or observations from staff/researchers, if available, on strengths and shortcomings of the application, especially if they point to unintended/unexpected effects or uses. This includes (if available) reasons for why people did or did not use the application as intended by the developers.

	1	2	3	4	5	
subitem not at all important	○	○	○	○	○	essential

Does your paper address subitem 19-ii?

Copy and paste relevant sections from the manuscript (include quotes in quotation marks "like this" to indicate direct quotes from your manuscript), or elaborate on this item by providing additional information not in the ms, or briefly explain why the item is not applicable/relevant for your study

您的回答

DISCUSSION

22) Interpretation consistent with results, balancing benefits and harms, and considering other relevant evidence

NPT: In addition, take into account the choice of the comparator, lack of or partial blinding, and unequal expertise of care providers or centers in each group

22-i) Restate study questions and summarize the answers suggested by the data, starting with primary outcomes and process outcomes (use)

Restate study questions and summarize the answers suggested by the data, starting with primary outcomes and process outcomes (use).

	1	2	3	4	5	
subitem not at all important	○	○	○	○	○	essential

Does your paper address subitem 22-i? *

Copy and paste relevant sections from the manuscript (include quotes in quotation marks "like this" to indicate direct quotes from your manuscript), or elaborate on this item by providing additional information not in the ms, or briefly explain why the item is not applicable/relevant for your study

The present study investigated the effects of exergame and aerobic exercise on executive function and physical function in older adults with dementia. We found a significant shorter flanker task RT in response to exergame training. Moreover, exergame generally yielded larger increases in neural activities that are related to attention and working memory, and greater enhancement in lower body muscle strength and cardiorespiratory endurance compared to aerobic exercise.

In terms of behavioral performance, shorter flanker task RT following exergame training in both congruent and incongruent signals in the present study indicates the greater facilitation of executive function. In our previous finding, both exergaming and treadmill exercise resulted in significantly shorter Stroop task RT in patients with metabolic syndrome through improvements in basic information processing and executive function suppression control [39]. Another study noted that combining physical and cognitive stimulation in 8 weeks of multimodal exercise substantially improved information processing speed in older adults [40]. Collectively, participating in aerobic exercise training improves information processing speed in older adults, regardless of the exercise intensity and type. Consistent with the results of these previous studies, the results of the present study showed that exergame training resulted in a significant improvement in information processing speed, suggesting that auditory, visual and motor sensory stimulation during exergaming could accelerate information processing speed in elderly people with dementia. Although there was no significant interaction between the group and the intervention in the present study, there was no significant change in flanker task RT by aerobic exercise group, so we cannot rule out the possibility that exergaming has a more beneficial effect on information processing speed than general aerobic exercise.

In the present study, exergame training significantly increased the congruent N2 amplitude in the Fz and Cz cortices, and reduced latency in the Cz cortex. Distinguishing stimulus and cognitive processes are closely related to attention during the tasks [41]. Hwang and colleagues found that reduced N2 activation in the central lobe and occipital cortex after acute aerobic exercise [42]. Similarly, decreased N2 amplitude and longer N2 latency were observed during exercise [43]. We previously reported increased N2 amplitude in response to exergame training, suggesting that exergame effectively increases selective attention by regulating the activities of the central and occipital cortex [39]. Performing exercise in a virtual environment also increased N2 amplitude and reduced latency [31]. These changes in brain activity reflect exercise with extra environmental stimulation promoted decision making (frontal and central areas) and visual perception (occipital areas). In this context, using cognition-stimulating games may facilitate the ability to clearly distinguish stimuli by activating the cerebral cortex with visual and auditory stimuli, thereby promoting cognitive processes. Therefore, our findings suggest that exergame improves attention by effectively promoting neuronal activity in older individuals with dementia. Furthermore, considering that in the present study, the N2 latency after exergame was shorter than that of aerobic exercise, and the congruent N2 amplitude and latency were not changed with the aerobic exercise, it is still possible that exergame may have a greater effect on neural activity related to executive function and attention compared to than a simple form of aerobic exercise.

We also found a greater increase in congruent and incongruent P3b amplitude with exergame than aerobic exercise in the present study. This suggests that exergame may have a greater effect on working memory than general aerobic exercise. Tsai and colleagues reported that performing resistance training for 12 months was associated with maintaining

[illegible] the capacities for allocating attention as measured by P3b amplitude in healthy older males [44]. Our previous investigation also found that aerobic exercise and resistance exercise have positive effects on neuronal activation, especially on the P3b amplitude in young adults [45]. Additionally, exergame improved categorization of the incoming information and updating the context of working memory as measured by P300 amplitude in patients with metabolic syndrome [39]. Taken together, both exercise and exergame induce brain activity through visual perceptual stimuli and activate nerve cells in the brain to promote information processing related to stimuli judgment and decision making. Extending on these results, the results of this study clearly indicate that exergame can be a more effective intervention in improving the classification of input information and working memory in the elderly with dementia compared to general aerobic exercise.
A recent meta-analysis showed that interventions combining both physical training and cognitive training improved cognitive function than physical training alone [46], and our previous study supports the results of the meta-analysis [39]. In turn, better executive function is thought to be supported by healthy lifestyle interventions, including physical activity [47]. Thus, the exergame intervention used in the present study would have increased the brain's ability to rapidly recognize consistent and inconsistent stimuli and simultaneously make judgements, faster than normal aerobic exercise. It is also important to note that exergame showed greater improvements in lower body muscle strength and cardiorespiratory endurance compared to aerobic exercise. During the "Alchemist Treasure" game in the ExerHeart@ device used in this study, participants had to continuously walk or adjust their body position. These constant body movements throughout the game may also have resulted in improvements in their cardiorespiratory endurance. Our results are in agreement with a previous study showing that exergame program effectively improved cardiopulmonary endurance and leg muscle strength in healthy middle-aged and older adults [48]. In addition, lower body strength is closely related to cardiopulmonary endurance [49], and aerobic exercise improves repetitive muscle contraction and cardiopulmonary endurance and enhances everyday abilities such as climbing stairs or sitting and standing up from a chair [50]. Therefore, exergame may be an effective exercise in improving the lower body muscle strength and cardiorespiratory endurance of older adults.

22-ii) Highlight unanswered new questions, suggest future research

Highlight unanswered new questions, suggest future research.

	1	2	3	4	5	
subitem not at all important	○	○	○	○	○	essential

Does your paper address subitem 22-ii?

Copy and paste relevant sections from the manuscript (include quotes in quotation marks "like this" to indicate direct quotes from your manuscript), or elaborate on this item by providing additional information not in the ms, or briefly explain why the item is not applicable/relevant for your study

您的回答

20) Trial limitations, addressing sources of potential bias, imprecision, and, if relevant, multiplicity of analyses

20-i) Typical limitations in ehealth trials

Typical limitations in ehealth trials: Participants in ehealth trials are rarely blinded. Ehealth trials often look at a multiplicity of outcomes, increasing risk for a Type I error. Discuss biases due to non-use of the intervention/usability issues, biases through informed consent procedures, unexpected events.

	1	2	3	4	5	
subitem not at all important	○	○	○	○	○	essential

Does your paper address subitem 20-i? *

Copy and paste relevant sections from the manuscript (include quotes in quotation marks "like this" to indicate direct quotes from your manuscript), or elaborate on this item by providing additional information not in the ms, or briefly explain why the item is not applicable/relevant for your study

The present study has some limitations. First, our study does not have a non-exercise control group, warranting some caution in the interpretation of the results. Nevertheless, pre-posttest designs are commonly used to assess the effectiveness of intervention over time, and our results are consistent with other exercise intervention studies that have shown significant interventional effects in older adults with dementia. Thus, it is not likely that our results simply reflect passage of time or other nonspecific intervention effects. Second, we had a relatively small number of participants (n=24) and homogeneous characteristic (e.g., all Asians). As a result, the statistical power may be lower in this study, but at least to compensate for this problem, the effect sizes were calculated as references. Finally, since our study is limited to using only a single exergame, it is necessary to verify the effectiveness of other games. Therefore, these limitations may need to be supplemented in future studies.

21) Generalisability (external validity, applicability) of the trial findings

NPT: External validity of the trial findings according to the intervention, comparators, patients, and care providers or centers involved in the trial

21-i) Generalizability to other populations

Generalizability to other populations: In particular, discuss generalizability to a general Internet population, outside of a RCT setting, and general patient population, including applicability of the study results for other organizations

	1	2	3	4	5	
subitem not at all important	○	○	○	○	○	essential

Does your paper address subitem 21-i?

Copy and paste relevant sections from the manuscript (include quotes in quotation marks "like this" to indicate direct quotes from your manuscript), or elaborate on this item by providing additional information not in the ms, or briefly explain why the item is not applicable/relevant for your study

您的回答

21-ii) Discuss if there were elements in the RCT that would be different in a routine application setting

Discuss if there were elements in the RCT that would be different in a routine application setting (e.g., prompts/reminders, more human involvement, training sessions or other co-interventions) and what impact the omission of these elements could have on use, adoption, or outcomes if the intervention is applied outside of a RCT setting.

	1	2	3	4	5	
subitem not at all important	○	○	○	○	○	essential

Does your paper address subitem 21-ii?

Copy and paste relevant sections from the manuscript (include quotes in quotation marks "like this" to indicate direct quotes from your manuscript), or elaborate on this item by providing additional information not in the ms, or briefly explain why the item is not applicable/relevant for your study

您的回答

OTHER INFORMATION

23) Registration number and name of trial registry

Does your paper address CONSORT subitem 23? *

Copy and paste relevant sections from the manuscript (include quotes in quotation marks "like this" to indicate direct quotes from your manuscript), or elaborate on this item by providing additional information not in the ms, or briefly explain why the item is not applicable/relevant for your study

The trial will be retroactively registered.

24) Where the full trial protocol can be accessed, if available

Does your paper address CONSORT subitem 24? *

Cite a Multimedia Appendix, other reference, or copy and paste relevant sections from the manuscript (include quotes in quotation marks "like this" to indicate direct quotes from your manuscript), or elaborate on this item by providing additional information not in the ms, or briefly explain why the item is not applicable/relevant for your study

The full protocol is not publicly available but can be requested through the corresponding author.

25) Sources of funding and other support (such as supply of drugs), role of funders

Does your paper address CONSORT subitem 25? *

Copy and paste relevant sections from the manuscript (include quotes in quotation marks "like this" to indicate direct quotes from your manuscript), or elaborate on this item by providing additional information not in the ms, or briefly explain why the item is not applicable/relevant for your study

The authors have no competing interests to report.

X27) Conflicts of Interest (not a CONSORT item)

X27-i) State the relation of the study team towards the system being evaluated

In addition to the usual declaration of interests (financial or otherwise), also state the relation of the study team towards the system being evaluated, i.e., state if the authors/evaluators are distinct from or identical with the developers/sponsors of the intervention.

	1	2	3	4	5	
subitem not at all important	○	○	○	○	○	essential

Does your paper address subitem X27-i?

Copy and paste relevant sections from the manuscript (include quotes in quotation marks "like this" to indicate direct quotes from your manuscript), or elaborate on this item by providing additional information not in the ms, or briefly explain why the item is not applicable/relevant for your study

您的回答

About the CONSORT EHEALTH checklist

As a result of using this checklist, did you make changes in your manuscript? *

- ○ yes, major changes
- ○ yes, minor changes
- ◉ no

What were the most important changes you made as a result of using this checklist?

您的回答

How much time did you spend on going through the checklist INCLUDING making changes in your manuscript *

5 hour did spend on going through the checklist INCLUDING making changes in manuscript

As a result of using this checklist, do you think your manuscript has improved? *

- ○ yes
- ◉ no
- ○ 其他:

Would you like to become involved in the CONSORT EHEALTH group?

This would involve for example becoming involved in participating in a workshop and writing an "Explanation and Elaboration" document

- ○ yes
- ○ no
- ○ 其他:

Any other comments or questions on CONSORT EHEALTH

您的回答

STOP - Save this form as PDF before you click submit

To generate a record that you filled in this form, we recommend to generate a PDF of this page (on a Mac, simply select "print" and then select "print as PDF") before you submit it.

When you submit your (revised) paper to JMIR, please upload the PDF as supplementary file.

Don't worry if some text in the textboxes is cut off, as we still have the complete information in our database. Thank you!

Final step: Click submit !

Click submit so we have your answers in our database!

提交 清除表单内容

切勿通过 Google 表单提交密码。

此内容不是由 Google 所创建，Google 不对其作任何担保。 举报滥用行为 - 服务条款 - 隐私权政策

Google 表单
